# Brawn before bite in endemic Asian eutherian mammals after the end-Cretaceous extinction

**DOI:** 10.7554/eLife.108917

**Published:** 2026-06-30

**Authors:** Z Jack Tseng, Qian Li, Suyin Ting

**Affiliations:** 1 Department of Integrative Biology and Museum of Paleontology, University of California, Berkeley Berkeley United States; 2 Department of Vertebrate Paleontology, Natural History Museum of Los Angeles County Los Angeles United States; 3 Key Laboratory of Vertebrate Evolution and Human Origins, Institute of Vertebrate Paleontology and Paleoanthropology, Chinese Academy of Sciences Beijing China; 4 Museum of Natural Science, Louisiana State University Baton Rouge United States; University of Neuchâtel Switzerland; University of Neuchâtel Switzerland

**Keywords:** Mammalia, Eutheria, Pantodonta, Paleocene, Nanxiong Basin, Other

## Abstract

The first 10 million years (Myr) following the Cretaceous-Paleogene (K-Pg) mass extinction marked a period of global greenhouse conditions and dramatic rise of placental mammals. Because ~80% of known terrestrial sections capturing post-K-Pg mammal recovery come from North America, a substantial knowledge gap exists in the tempo and mode of recovery in Asia, where only 3% of global sites are located and most contain species found nowhere else. We show that isolated Paleocene eutherian assemblages from China (1) exhibited high mean tooth size and disparity early in the Paleocene, (2) shifted in their dental shape in parallel with regional and global environmental changes later in the Paleocene, and (3) achieved maximum dental shape-performance covariation near the end of the first 10 Myr post-K-Pg. This ‘brawn before bite’ transformation, coupled with prolonged dental shape versus performance variability, favors a scenario whereby many living orders of eutherian mammals were borne out of phenotypically and functionally plastic ancestral assemblages, including those in tropical South China, during the Paleocene.

## eLife digest

Over the course of Earth's history, five major mass extinctions have shaped life as we know it today. The most recent occurred around 66 million years ago at the end of the Cretaceous Period, leading to the demise of non-avian dinosaurs and ushering in the age of mammals.

Most of what we know about how mammals recovered from this mass extinction comes from fossil sites in North America. Far less is known about how ancient mammals in other parts of the world adapted to a dramatically altered environment after the disappearance of large dinosaurs.

Tseng, Li and Ting investigated how some of the earliest mammal species in Asia recovered following this global mass extinction. They studied 200 individual teeth from 48 specimens of extinct mammals, including members of the Pantodonta (large herbivorous mammals), Arctostylopidae (stocky herbivorous and omnivorous mammals), and Anagaloidea (a group closely related to rodents). Using high-resolution 3D models of these rare fossil teeth, the researchers quantified variation in tooth shape and function.

Their analyses revealed that mammals in East Asia, particularly South China, were already relatively large during the early Palaeocene, the first epoch of the age of mammals. Over the next five million years, their teeth became increasingly specialised for different forms of chewing and food processing, reflecting the likely diversification of diets and ecological niches within mammal communities. The findings also suggest that these early mammals were ecologically flexible, meaning they were able to exploit a wide range of food resources and adapt to changing environmental conditions. As ecosystems recovered and transformed during the first 10 million years after the end-Cretaceous mass extinction, the relationship between tooth shape and function also shifted, indicating changing evolutionary pressures and ecological opportunities.

The study by Tseng, Li and Ting helps fill an important gap in our understanding of how biodiversity recovered in different regions of the world following the most recent major mass extinction. Their findings may also inform predictive models and conservation strategies aimed at understanding how modern animals could respond to future biodiversity crises. Insights from the past can help us prepare for the present and future, as rapid climate and environmental change increasingly challenge the coexistence of humans and the natural world.

## Introduction

The Cretaceous-Paleogene (K-Pg) mass extinctions accelerated the formation of modern-day global biota. In particular, the more than 6000 species of living placental mammals trace their origins to the diversification of major orders around or after the K-Pg boundary (Foley et al., 2023; Liu et al., 2017; O’Leary et al., 2013). Different hypotheses (e.g. early rise, suppression, and late rise) about the timing of their adaptive radiation have been proposed (Benevento et al., 2023; Grossnickle et al., 2019; O’Leary et al., 2013). Regardless of the diversification scenarios favored by the competing explanations, the Paleocene epoch (66–56 Myr ago) has been highlighted as among the most critical time intervals for establishing the macroevolutionary parameters of the placental adaptive radiation (Halliday et al., 2017; Upham et al., 2021) (but see Grossnickle et al., 2019). However, substantial asymmetry exists in the quantity and quality of terrestrial fossil records that document the first 10 Myr of the ‘Age of Mammals’, with ~80% of known terrestrial K-Pg boundary sections worldwide occurring in North America (Vajda and Bercovici, 2014). Earliest Paleocene mammals are not known from Europe (Csiki-Sava et al., 2015; De Bast and Smith, 2017), rendering detailed reconstructions of post-K-Pg recovery in that continent difficult. The post-K-Pg fossil assemblages from Asia have hitherto not been considered in analyses of post-K-Pg recovery dynamics (Wang et al., 2007).

The North American fossil record suggests that post-K-Pg eutherian taxonomic diversity recovery was relatively rapid, most occurred within the first ~0.3–1 Myr of the Paleocene epoch (Alroy, 1999; Grossnickle and Newham, 2016; Longrich et al., 2016; Lyson et al., 2019; Wilson, 2014; Wing et al., 1995). This initial eutherian diversification was driven by archaic groups (i.e. stem placental/eutherian lineages and extinct placental subgroups), followed by the first appearance of many modern orders during two peak hyperthermal events in the past 66 Myr, at the end-Paleocene and early Eocene climatic maxima, respectively (Bowen et al., 2002; Gingerich, 2010).

Distinct from these taxonomic recovery patterns, high selectivity of mammalian ecomorphological extinction across the K-Pg boundary indicates a primary productivity filter at the start of the Cenozoic Era (66 Myr ago to the present) (Wilson, 2013). Additionally, the first 10 Myr of mammalian brain evolution after the K-Pg was marked by niche partitioning along a size gradient; endocranial traits reflecting more complex sensory processing did not appear until the Eocene (56 Myr ago). This phenomenon has been termed the ‘brawn before brains’ hypothesis (Bertrand et al., 2022). A similar pattern in initial size-driven diversification followed by expansion of ecomorphological disparity is also observed in mammal jaws, indicating a general dynamic across phenotypic systems (Benevento et al., 2019). Whether this mode of evolution, whereby size disparity increase precedes other ecomorphological traits, should be understood as a global phenomenon during the post-K-Pg placental radiation remains untested, in large part because no such analyses have centered on non-North American continental fossil mammal records.

A major challenge with expanding analyses of post-K-Pg recovery to Paleocene mammal assemblages elsewhere in the world is the stratigraphically limited nature of early Cenozoic sequences that produce fossil mammals. In Asia, Paleocene localities in China represent the best studied to date (Wang et al., 2007). From the earliest Paleocene, highly regional and endemic faunas are known from a handful of sedimentary basins (Figure 1—figure supplement 1A). Among the recorded faunal elements, only the archaic placental clades Anagalida and Pantodonta are consistently sampled across the major subdivisions of the Paleocene (Wang et al., 2007) . Additional complications with ecomorphological analysis of these stem eutherians include uncertainty in their dietary ecology, having diverged prior to the crown radiation, and uncertainty in phylogenetic positions of Paleocene taxa (Halliday et al., 2017); thus, they are beyond the reach of conventional phylogenetic bracketing approaches to dietary reconstruction. Phenomic analysis of the placental radiation supports insectivory as the ancestral diet of the hypothetical placental ancestor, but uncertainty in the post K-Pg availability of insects and plants in some regions leaves some doubt as to the accuracy and scope of this ancestral state reconstruction (O’Leary et al., 2013). Herein, we treat the archaic Paleocene taxa in our analyses as having uncharacterized diets rather than categorizing them as insectivores, herbivores, or carnivores.

**Figure 1.**
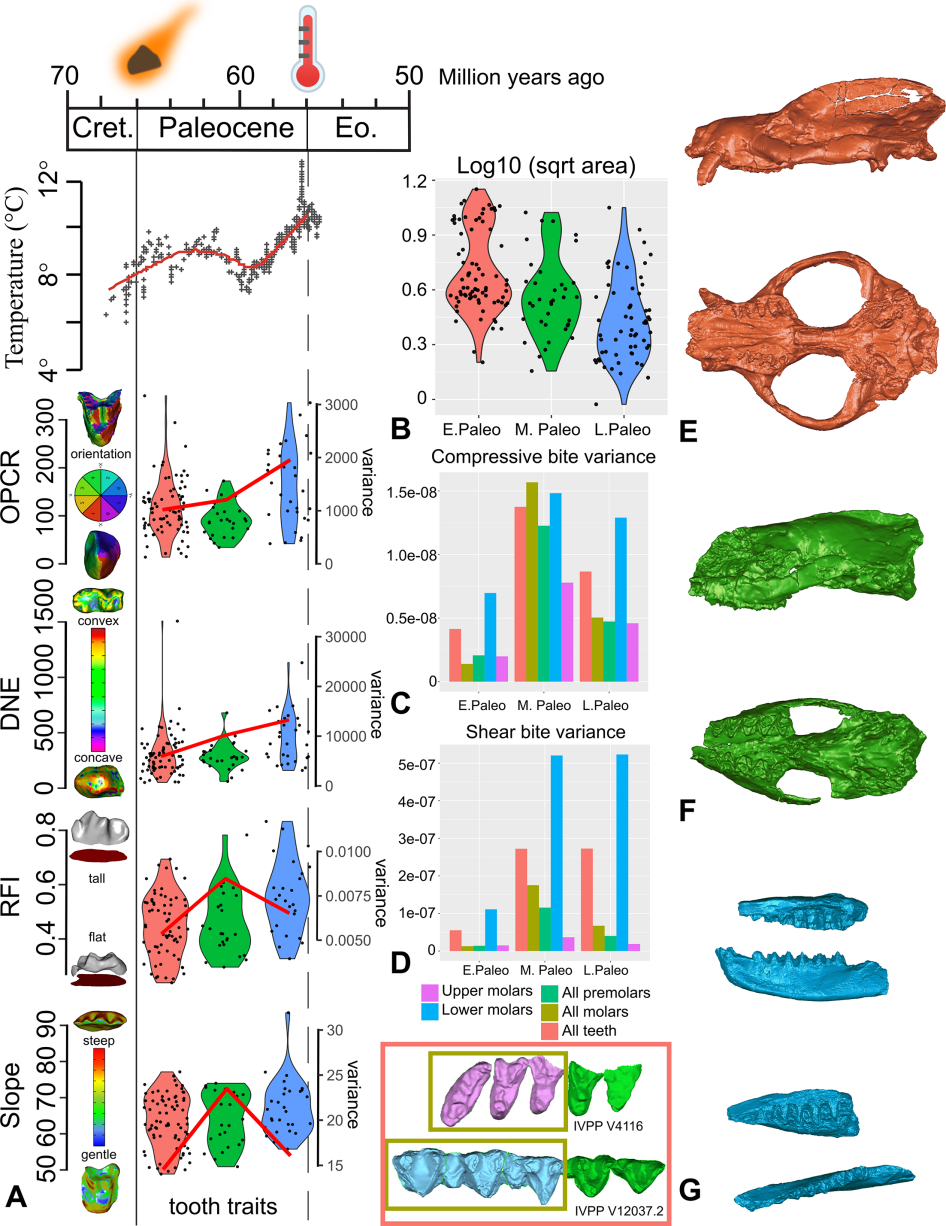
Temperature and fossil eutherian mammal dental trait shifts during the first 10 million years (Myr) of the Cenozoic. (**A**) Dental topographic trait values (boxplots) and mean variance (red curves) during the first 10 Myr of the Cenozoic, signifying the time after the Cretaceous-Paleogene (K-Pg) mass extinctions and before the Paleocene-Eocene hyperthermal event. Global temperature curve based on Zachos et al., 2001. Dental traits measured include crown complexity (OPCR, orientation patch count rotated), curvature (DNE, Dirichlet normal energy), height (RFI, relief index), and slope. (**B**) Eutherian tooth size distributions represented by log 10 square root tooth area, in units of log10 millimeters. (**C**) Variance of compressive bite performance based on tooth crown finite element simulations, in units of squared Joules. (**D**) Variance of shear bite performance based on tooth crown finite element simulations, in units of squared Joules. Examples of endemic Asian fossil specimens analyzed. (**E**) Lateral and ventral views of early Paleocene Chinese endemic pantodont (CEP) *Bemalambda nanhsiungensis* IVPP (Institute of Vertebrate Paleontology and Paleoanthropology, Chinese Academy of Sciences) V4116. (**F**) Lateral and ventral views of middle Paleocene CEP *Harpyodus decorus* IVPP 5035.1. (**G**) Lateral and occlusal views of late Paleocene CEP *Guichilambda zhaii* IVPP V12037.2 (dentary) and V12037.3 (maxillary fragment). Firey asteroid symbols indicate the end-Cretaceous asteroid impact in the Yucatán Peninsula; thermometer symbols indicate the Paleocene-Eocene hyperthermal event. Subdivisions of the Paleocene approximately correspond to the Shanghuan, Nongshanian, and Gashatan Asian Land Mammal Ages, respectively (see supplemental text for competing age boundary scenarios).

**Figure 2.**
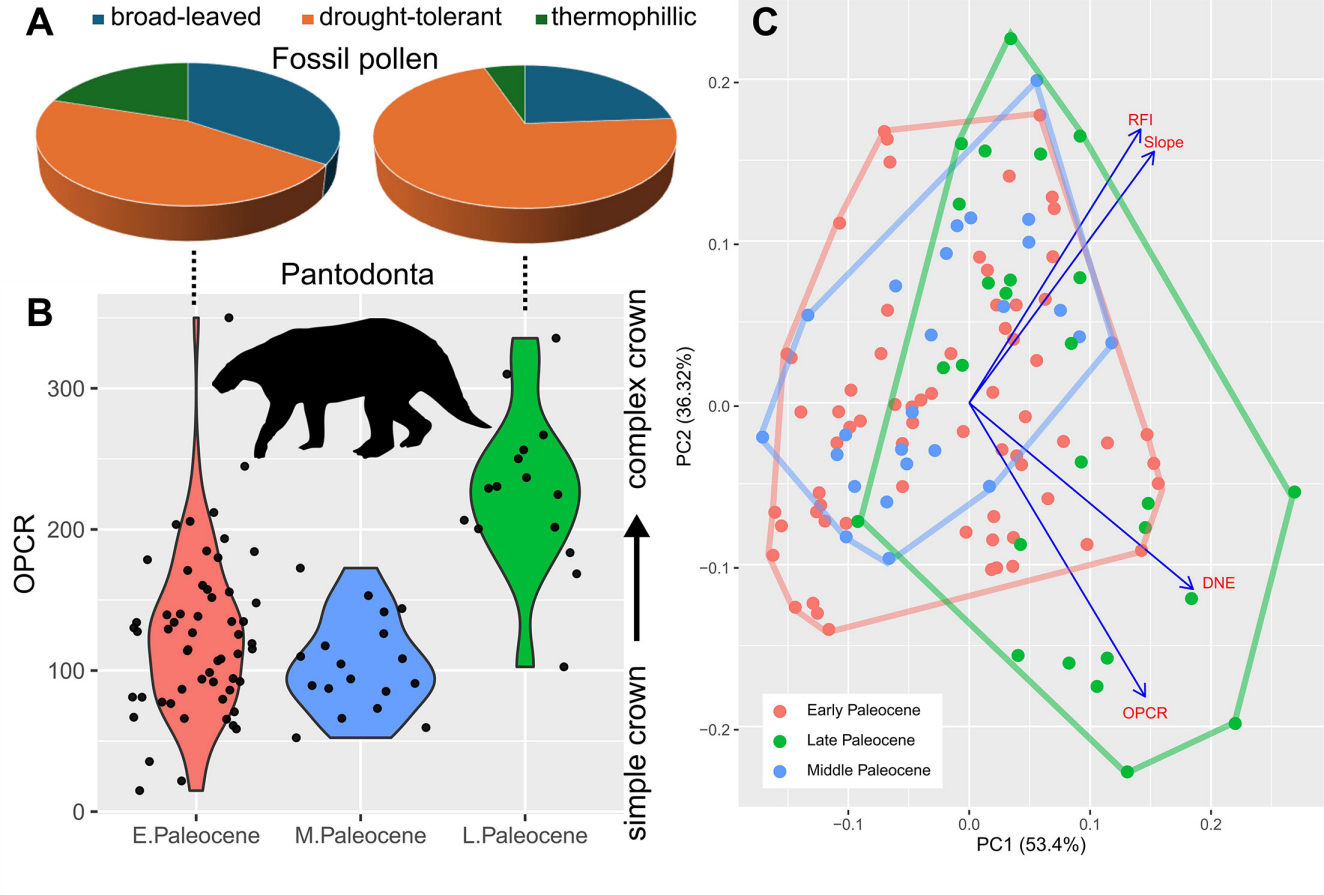
Association of paleopalynological data from the Nanxiong Basin, South China, and late Paleocene niche expansion in endemic Asian fossil eutherians. (**A**) Proportion of environmental humidity indicator taxa from early versus late Paleocene paleobotanical localities, respectively, in the Nanxiong Basin; data based on Ma et al., 2012; Xie et al., 2020 (Source data 10). (**B**) Boxplots of dental complexity (OPCR, orientation patch count rotated) in the Chinese endemic pantodont (CEP) data partition across the three Paleocene time intervals examined. Note the concomitant increase in CEP tooth complexity (OPCR) and increased proportion of drought-tolerant plant species in the Nanxiong Basin during the late Paleocene. (**C**) Principal component morphospace of all tooth data analyzed; convex hulls delineate overall morphospace occupation during each time interval. Eigenvectors of the four dental topographic traits are indicated in blue. Late Paleocene shift and expansion in dental topographic morphospace is statistically significant at the p=0.05 level (Table 1). Pantodont silhouette by S Traver from phylopic.org.

We investigated the timing of ecomorphological diversification by developing and leveraging the largest dataset to date of Paleocene Asian eutherian assemblages. Our analyses focused on eutherians from three of the most fossiliferous and biogeographically isolated Paleocene sedimentary sequences in paleotropical Asia: the Nanxiong, Qianshan, and Chijiang Basins in present-day South China (Chow et al., 1977; Clyde et al., 2008; Clyde et al., 2010; Ting et al., 2011; Wang et al., 2016; Figure 1—figure supplement 1). We generated a new phenotypic dataset of 200 Asian Paleocene eutherian teeth using high-resolution microcomputed tomography and laser scanning, capturing 37 species endemic to low-latitude east Asia and which are brought to bear on K-Pg recovery dynamics for the first time (Source data 1 and 2). Teeth are among the most well-preserved parts of fossil mammals, and the fact that they interface directly with the environment through mastication makes them suitable elements for studying potential ecology-morphology linkages. We used dental topographical traits as indicators of ecomorphological diversity (Lucas et al., 2008) and examined temporal shifts in tooth crown complexity, curvature, and height. We additionally assessed the association of topographical traits with tooth crown mechanical performance in terms of deformation resistance using topographic and simulation analyses. Our dataset spans the Paleocene, the first 10 Myr of the Cenozoic, enabling us to test the hypothesis that dental topography and tooth puncturing and shearing performance linkages showed delayed niche expansion relative to mean body size and size disparity during this initial period of post-K-Pg eutherian recovery in Asia (Figures 1 and 2).

**Figure 1—figure supplement 1.**
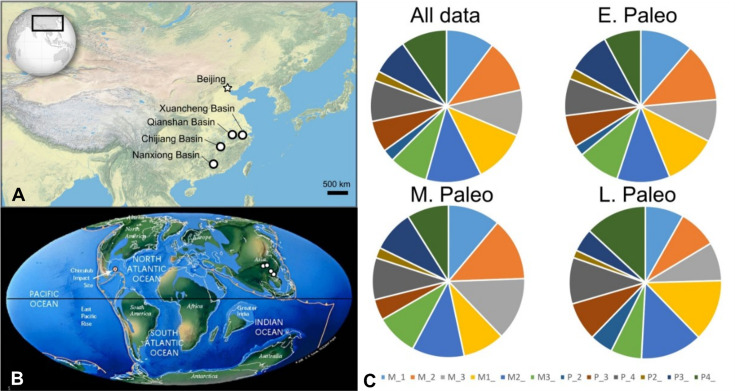
Maps of the fossil locality areas and proportions of teeth analyzed. The Paleocene sedimentary basins sampled are indicated on a modern satellite image (**A**) and on a paleogeographic map of the Cretaceous-Paleogene (K-Pg) transition (**B**). Satellite map from NASA (https://www.nasa.gov/) and paleogeographic map from C Scotese (https://www.scotese.com/) (Scotese, 2001). (**C**) Proportions of tooth positions present in data partitions. M_1-M_3, lower first to third molars; M1_-M3_, upper first to third molars; P_2-P_4, lower second to fourth premolars; P2_-P4_, upper second to further premolars; E, early, M, middle, L, late. The middle Paleocene partition has no P_2 sampled, but otherwise the proportions of tooth positions present in each time interval are generally similar to each other.

**Figure 1—figure supplement 2.**
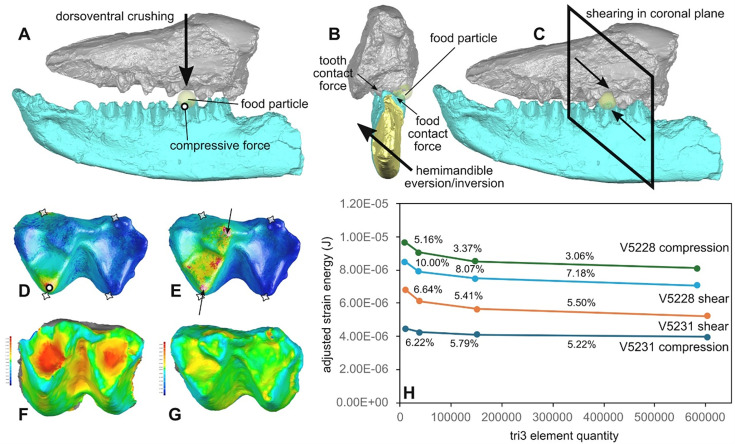
Finite element modeling boundary conditions and convergence tests. (**A**) Depiction of compressive/crushing force imposed on a lower molar cusp in a specimen of *G. zhaii* (IVPP V12037) in lateral view. (**B**) Depiction of shearing motion during hemimandible eversion/inversion cycle in coronal view. (**C**) Depiction of shearing motion in lateral view. (**D**) Boundary conditions of compressive bite simulations on the lower first molar of *Altilambda tenuis* (IVPP V5231). (**E**) Boundary conditions of shearing bite simulations on *A. tenuis*. Nodal constraints are indicated by stars, compressive force by circle, and shearing forces by black arrows. (**F**) Three-dimensional deviation map of the first lower molar of *Altilambda pactus* (IVPP V5228) comparing geometric difference between models built from original versus cast-derived image data; positive (red) and negative (blue) deviation maxima ±0.58 mm. (**G**) Three-dimensional deviation map of the first lower molar of *A. tenuis* (IVPP V5231), positive (red) and negative (blue) deviation maxima ±0.39 mm. (**H**) Convergence test of adjusted strain energy values (in Joules) of compression and shear bite simulations in IVPP V5228 and V5231. Percentage values indicate differences between adjacent model quantities (measured in number of three-noded triangular [tri3] elements). All model values at the lowest quantity models are considered converged based on a ≤10% threshold criterion.

**Figure 1—figure supplement 3.**
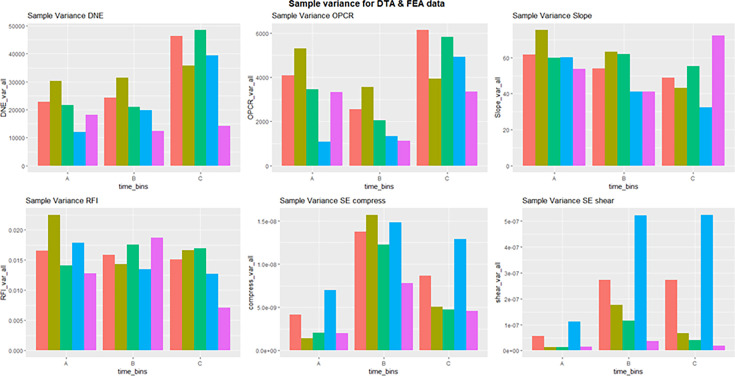
Sample variance (in squared units of each metric) of dental topographic metrics and dental performance traits for the overall dataset. Time bins refer to the early Paleocene (Bin A), middle Paleocene (Bin B), and late Paleocene (Bin C). Color bars represent different data partitions: all data (salmon), all molars (teal), all premolars (green), lower molars (blue), and upper molars (purple).

**Figure 1—figure supplement 4.**
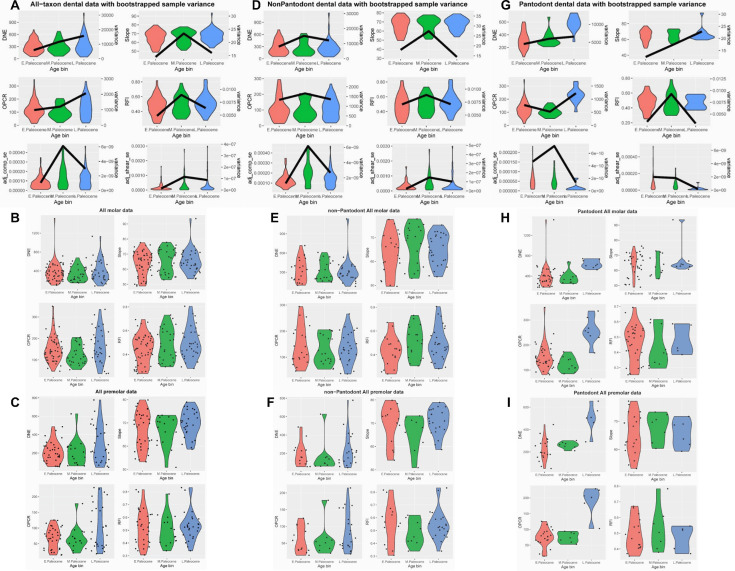
Boxplots of dental topographic and finite element simulated traits, related to Table 1. Dark lines in panels **A**, **D**, and **G **denote sample variance per million years, calculated from 1000 bootstrap samples of trait values pulled from uniform distributions within their estimated uncertainty ranges. The variance magnitudes are scaled to the duration of the Asian land mammal ages representing each time interval. (**A**) Dental topographic analysis (DTA) (DNE, OPCR, Slope, RFI) and finite element analysis (FEA) (compressive bite strain energy, shear bite strain energy) boxplots representing all tooth positions in the all-taxon partition. (**B**) DTA metrics for the molar partition of all taxa. (**C**) DTA metrics for the premolar partition of all taxa. (**D**) DTA and FEA boxplots of all tooth positions in the non-pantodont data partition. (**E**) DTA metrics for molars in the non-pantodont data partition. (**F**) DTA metrics for premolars in the non-pantodont data partition. (**G**) DTA and FEA boxplots of all tooth positions in the Chinese endemic pantodont (CEP) partition. (**H**) DTA metrics for molars in the CEP partition. (**I**) DTA metrics for premolars in the CEP partition. OPCR, orientation patch count rotated; DNE, Dirichlet normal energy; RFI, relief index.

**Figure 1—figure supplement 5.**
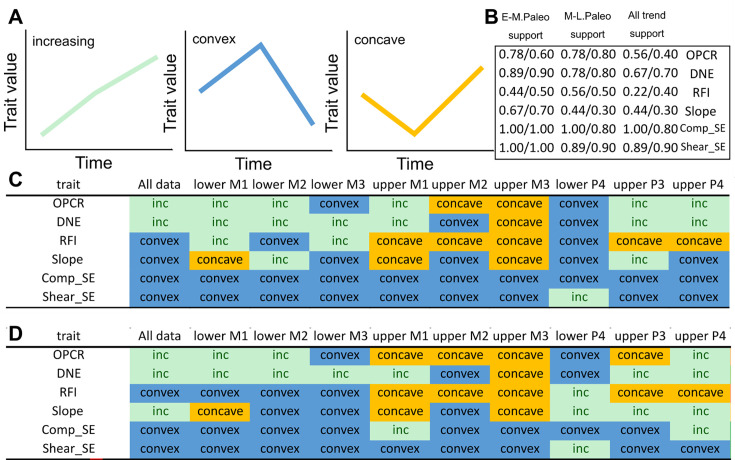
Trends through time for dental topographic analysis (DTA) and finite element analysis (FEA) traits by tooth position, related to Figure 1. (**A**) Categories of major trends observed through the three Paleocene time intervals. (**B**) Proportion of individual tooth position analyses that support the trend observed in the overall dataset. The first set of values is per-tooth variance; the second set of values is from pooled sample and per-tooth range lengths. (**C**) Individual tooth position variance trends in DTA and FEA trait values. (**D**) Individual tooth position range length trends in DTA and FEA trait values.

**Figure 1—figure supplement 6.**
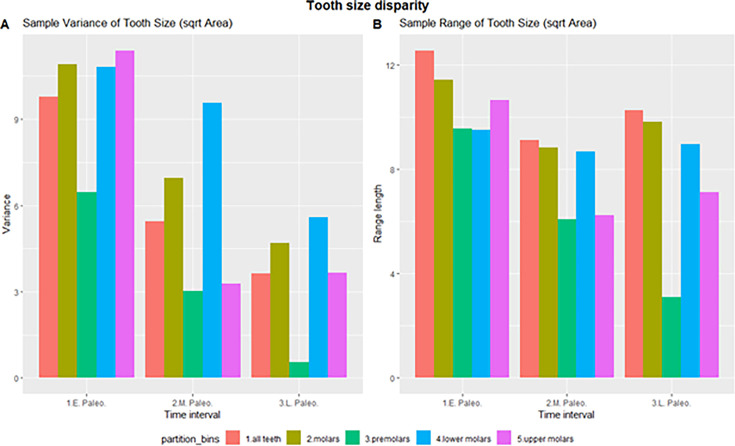
Tooth size disparity bar plots, related to Figure 1. (**A**) Variance of tooth size (sqrt of 2D tooth area) in different data partitions. (**B**) Range length of tooth size (sqrt of 2D tooth area) in different partitions.

## Results and discussion

### Summary of levels of topography-performance associations

**Figure 3.**
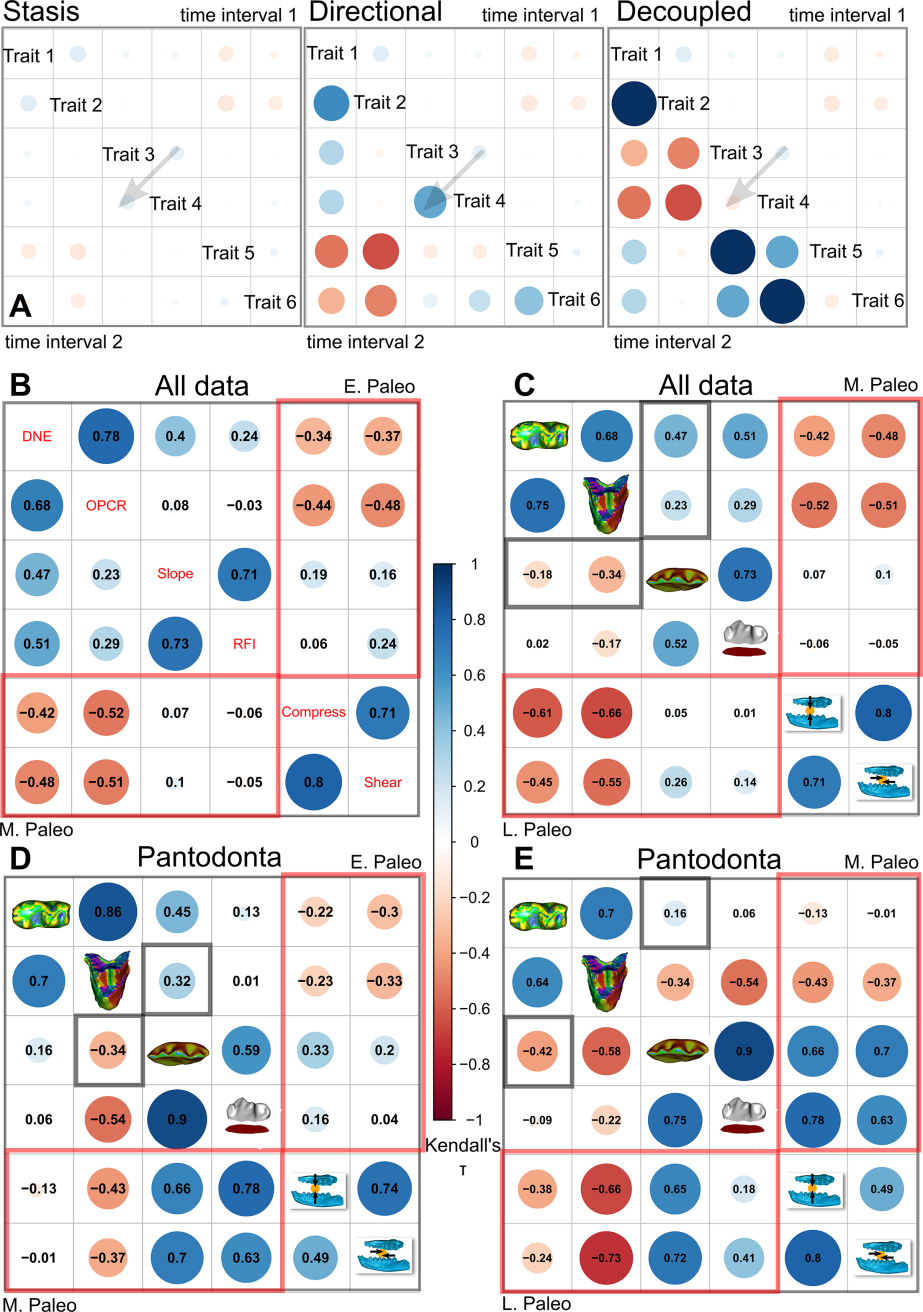
Correlation plots of dental topographic and bite performance traits in endemic Asian Paleocene eitherians. (**A**) Hypothetical correlation scenarios used to interpret stasis, directional, versus decoupled change through time in specimen data. (**B**) Pairwise ranked correlation coefficient estimated using Kendall’s τ between early and middle Paleocene dental topographic and performance traits in the main dataset. (**C**) Correlation between middle and late Paleocene traits in the main dataset. (**D**) Correlation between early and middle Paleocene traits in the Chinese endemic pantodont (CEP) data partition. (**E**) Correlation between middle and late Paleocene traits in the CEP data partition. Topography-performance correlations are marked in red boxes. Decoupled/reversed trait correlations are marked in gray boxes.

Correlation plot patterns (Figure 3): We performed correlation plot comparisons on combined DTA and FEA data, using Kendall’s τ as a measure of the strength of correlation. Contingency tables show that Dirichlet normal energy (DNE)/orientation patch count rotated (OPCR) to Compressive SE/Shear SE correlations increased from early to middle, then late Paleocene (Figure 3). Compared to the steady increase in integration shown by two-block partial least squares (2B-PLS) analyses (see below), this suggests that dental complexity and convexity were disproportionately driving the overall integration patterns, whereas cusp height and sharpness were very weakly associated with compressive and shear strain energy values.

**Figure 3—figure supplement 1.**
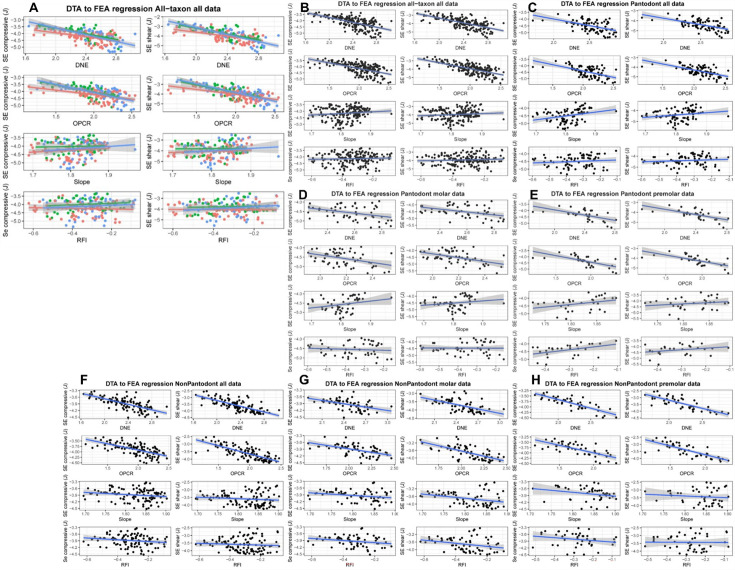
Linear regression analysis of dental topographic and bite performance datasets across different data partitions, related to Figure 3. (**A**) All-taxon all-teeth data partition; data points are colored according to early (red), middle (green), and late (blue) Paleocene age of the specimens. (**B**) All-taxon all-teeth data partition without time bin groups. (**C**) Chinese endemic pantodont (CEP) all-teeth data partition. (**D**) CEP molar data partition. (**E**) CEP premolar data partition. (**F**) Non-pantodont all-teeth partition. (**G**) Non-pantodont molar partition. (**H**) Non-pantodont premolar partition. Performance variables estimated using finite element analysis include tooth crown strain energy under compressive (left column within each panel) and shear bite (right column within each panel) simulations, respectively. Rows within panels represent different DTA metrics (from top to bottom: DNE, OPCR, Slope, RFI). Fitted regression lines are shown in blue; 95% confidence envelopes are shown in gray shades. OPCR, orientation patch count rotated; DNE, Dirichlet normal energy; RFI, relief index.

**Figure 3—figure supplement 2.**
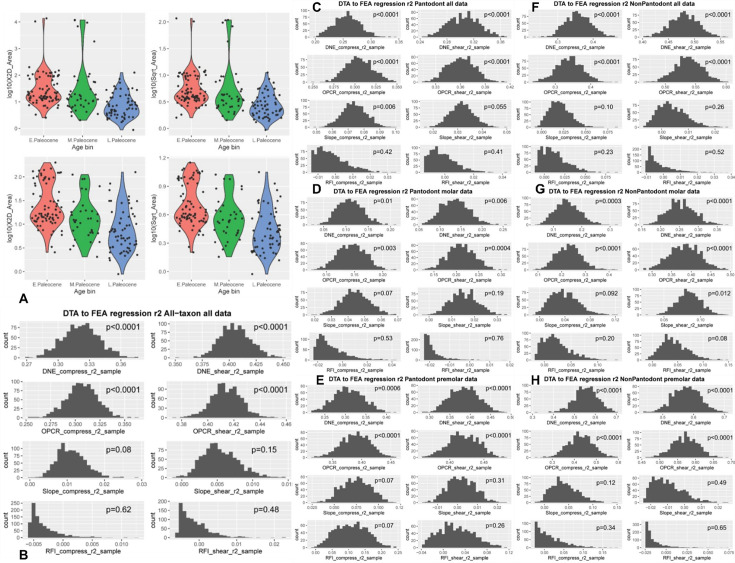
Tooth size boxplots and adjusted r*^2^* distributions from dental topographic analysis (DTA)-finite element analysis (FEA) linear regression analyses, related to Figure 3. (**A**) Boxplots of tooth size by 2D area (left column within panel) or square root of 2D area (right column within panel). The top row within panel A includes sampling outliers (very large Chinese endemic pantodonts [CEPs]), bottom row excludes outliers. The larger CEPs are removed in the bottom plots in order to more fully display the distribution of tooth sizes across the bimodal distribution of sizes in the main data cluster in each time bin, respectively. (**B**) Distribution of adjusted r*^2^* values from linear regression analysis of dental topographic and dental performance datasets for all-taxon all-teeth data partition. (**C**) CEP all-teeth partition. (**D**) CEP molar partition. (**E**) CEP premolar partition. (**F**) Non-pantodont all-teeth partition. (**G**) Non-pantodont molar partition. (**H**) Non-pantodont premolar partition. Adjusted r*^2^* values were generated from 1000 bootstrap samples of Dirichlet normal energy (DNE) and FEA trait values for each specimen from uniform distributions that incorporate uncertainty in DNE and FEA value estimates. p-Values were calculated from t-test of bootstrap sample p-values against the hypothesis of p≥0.05.

**Figure 3—figure supplement 3.**
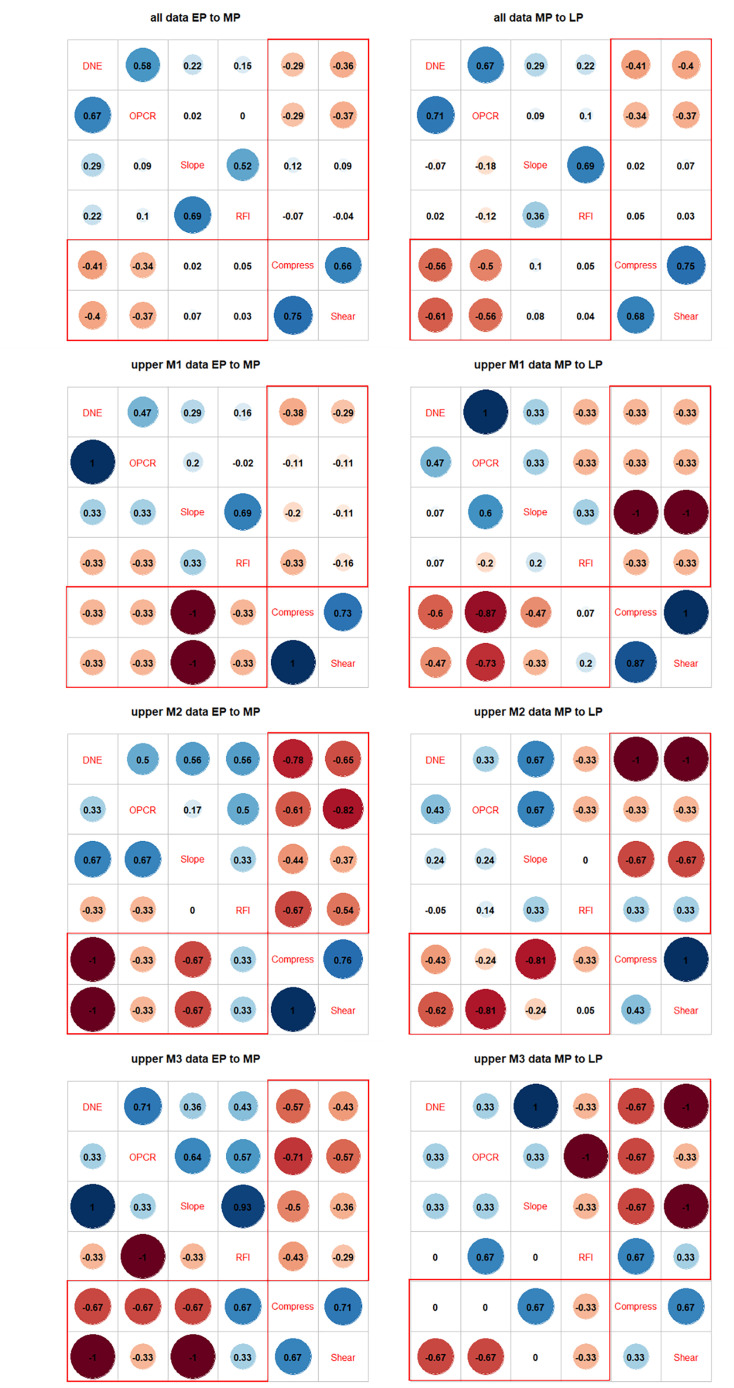
Correlation through time plots for upper molar dental topographic analysis (DTA) and finite element analysis (FEA) data, related to Figure 3. The upper diagonal in the left column shows early Paleocene (EP) correlations, and the lower diagonal in the left column shows middle Paleocene (MP) correlations. The upper diagonal in the right column shows middle Paleocene correlations, and the lower diagonal in the right column shows late Paleocene (LP) correlations. Red boxes indicate correlations between DTA and FEA traits. Dark gray boxes indicate decoupling of correlation directions across time intervals. Upper M1 patterns generally reflect the trend recovered from analysis of the overall dataset, but M2 and M3 results display inconsistent DTA-FEA correlations, possibly due to small sample sizes.

**Figure 3—figure supplement 4.**
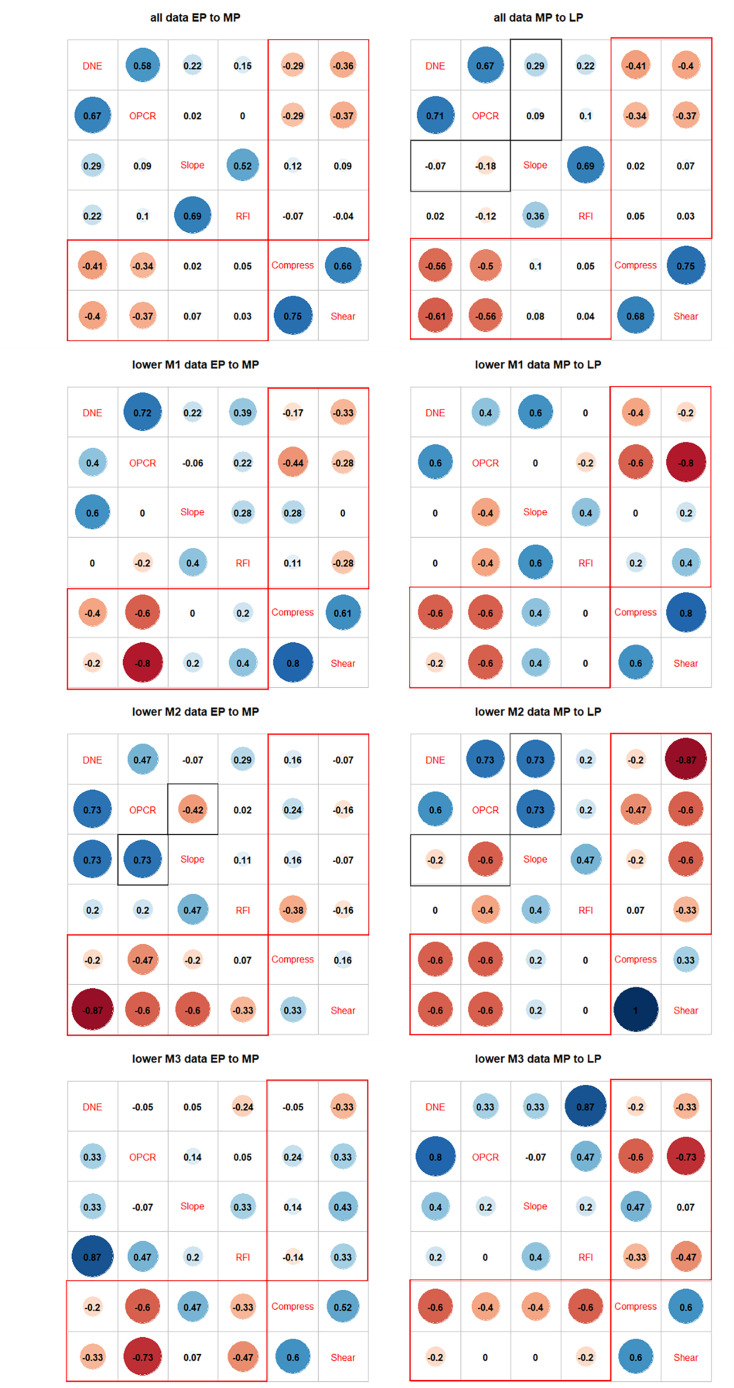
Correlation through time plots for lower molar dental topographic analysis (DTA) and finite element analysis (FEA) data, related to Figure 3. The upper diagonal in the left column shows early Paleocene (EP) correlations, and the lower diagonal in the left column shows middle Paleocene (MP) correlations. The upper diagonal in the right column shows middle Paleocene correlations, and the lower diagonal in the right column shows late Paleocene (LP) correlations. Red boxes indicate correlations between DTA and FEA traits. Dark gray boxes indicate decoupling of correlation directions across time intervals. Lower molar patterns generally replicate those recovered in the overall analyses, but lower M1 and M2 signals appear to be stronger than those for lower M3.

**Figure 3—figure supplement 5.**
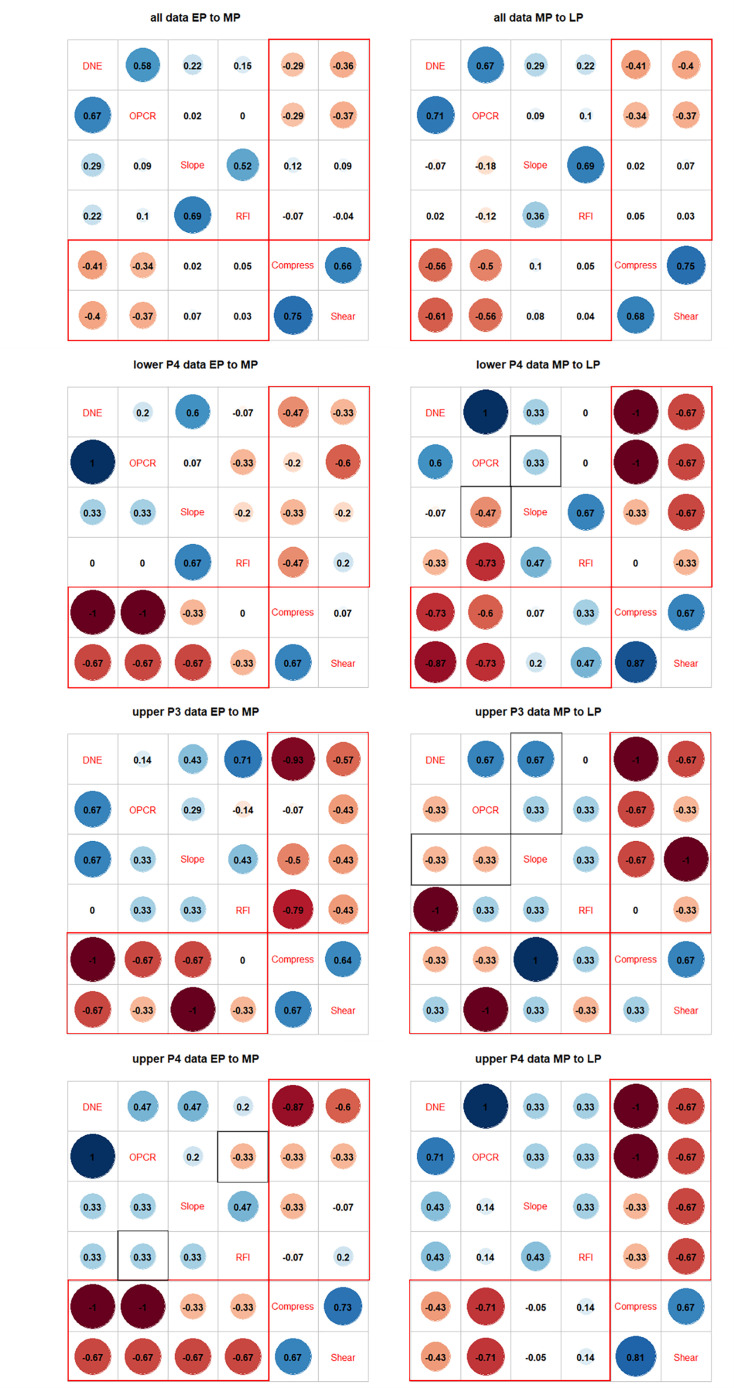
Correlation through time plots for premolar dental topographic analysis (DTA) and finite element analysis (FEA) data, related to Figure 3. The upper diagonal in the left column shows early Paleocene (EP) correlations, and the lower diagonal in the left column shows middle Paleocene (MP) correlations. The upper diagonal in the right column shows middle Paleocene correlations, and the lower diagonal in the right column shows late Paleocene (LP) correlations. Red boxes indicate correlations between DTA and FEA traits. Dark gray boxes indicate decoupling of correlation directions across time intervals. Low sample sizes make premolar correlations unstable, with general pattern showing EP-MP strengthening followed by MP-LP stasis or weakening.

We additionally conducted correlation analyses across time using data partitions representing individual tooth positions (Figure 3—figure supplements 3–5) to verify the overall trend observed using the total dataset (Figure 3). Upper M1 patterns generally reflect the trend recovered from analysis of the overall dataset, but M2 and M3 results display inconsistent DTA-FEA correlations, possibly due to small sample sizes. Lower molar patterns generally replicate those recovered in the overall analyses, but lower M1 and M2 signals appear to be stronger than those for lower M3. Finally, low sample sizes make premolar-specific correlations unstable, with general patterns showing EP-MP strengthening followed by MP-LP stasis or weakening.

Per-time variance patterns (Figure 1—figure supplements 3 and Figure 1—figure supplement 5) : The overall dataset showed a steady increase in DNE and OPCR variance, differing from the middle Paleocene peak pattern seen in the relief index (RFI), Slope, Compressive SE, and Shear SE variances. The non-pantodont partition shows a more consistent pattern between DTA and FEA variances, with all traits showing a middle Paleocene spike. Lastly, the CEP data partition shows even more mosaic patterns than the overall data partition. CEP DNE, OPCR, and Slope show variance increases over time, whereas Slope and Compressive SE exhibit a middle Paleocene spike. Shear SE shows a steady decline in variance over time. The disaggregated molar and premolar data partitions do not contain large sample sizes; thus, we caution against over-interpreting the finer patterns until a more statistically robust sample can be analyzed. The per-tooth position analyses, although broadly supporting the early-middle Paleocene trends for both DTA and FEA data (and for FEA data in general across all time intervals), provide lower support for the middle-late Paleocene trends. Support is lowest when RFI and Slope values are analyzed by tooth position. In addition to the smaller sample sizes included in these partitioned analyses, this result may indicate that different tooth positions may record somewhat different, sometimes opposing, DTA signals but consistent FEA signals. This suggests structure-performance decoupling at the tooth position level. Similar differences between the pooled sample results and per-tooth trends when using range length (maximum trait value – minimum trait value for a given data partition) are recovered (Figure 1—figure supplement 5B and D).

Trait correlation patterns (Figure 3—figure supplements 1 and 2): To more precisely examine the relationships between pairs of DTA and FEA traits, we performed pairwise linear regression analyses using bootstrapped sample estimates of trait values. The outputs show that the 2B-PLS results below are mainly driven by associations between higher DNE and OPCR with lower Compressive and Shear SE (Figure 1—figure supplement 5). This association is particularly strong in premolar partitions and less strong in molar partitions (Figure 3—figure supplement 1), and doesn’t appear to represent differences between CEPs and non-pantodonts overall. When examined through time, there does seem to be a difference in how strongly DTA-FEA are associated; the spikes in Compressive and Shear SE values in the middle Paleocene time bin are not mirrored in DNE and OPCR values in the same time interval, suggesting a breakdown during middle Paleocene in the DTA-FEA relationship established in the early Paleocene. This result specifically mirrors findings from the CEP all-teeth data partition and non-pantodont molar partition 2B-PLS analysis (see below).

2B-PLS patterns (Table 2): The overall dataset exhibited a steady increase in r coefficient values from the early to the late Paleocene, indicating strengthening DTA-FEA integration over time. The CEP data partition showed a dip in integration in the middle Paleocene in the all-teeth partition, a steady decrease in the premolar partitions, but the opposite pattern (a spike) in the molar partition. Non-pantodonts show a different pattern, where all-teeth and premolar partitions spike in integration during the middle Paleocene, whereas the molar partition dips in the middle Paleocene.

### Sensitivity analyses of tooth size trends

Tooth disparity was highest during the early Paleocene time interval of our dataset; this is consistent both using variance and range length as the disparity measure (Figure 1—figure supplement 6). The overall decrease in size disparity and variance across the Paleocene based on the total dataset was additionally tested in individual tooth position partitions (Table 4). The overall disparity trends are also observed in premolar and upper molar data partitions, whereas overall tooth size trends are observed mainly in the lower premolar 4 data partition. Decrease in mean tooth size is most consistently observed across multiple tooth partitions for the early Paleocene to late Paleocene comparison (and less clear-cut for early-middle and middle-late Paleocene comparisons, respectively). Shifts during the middle Paleocene are variably supported depending on tooth position analyzed. Given that the middle Paleocene is represented by the smallest sample size, the uncertainty surrounding per-tooth position trends during this time period may be explained by the low sample available for this study.

### Sample and methodological limitations

The highly fragmentary nature of early Cenozoic mammal fossils in Asia means that even the best preserved faunas studied herein contain substantial missing information. First, the absence of a high-resolution chronological framework prevents the fossil data from being analyzed on a continuous time axis; binning the samples into three main intervals within a 10 Myr period hinders additional hypotheses about the environmental and climatic correlations of the dental structure-performance results presented. Second, the uneven sampling of the available mammalian assemblage throughout the Paleocene sites in China limits the breadth of ecomorphological categories included in the analyses; rarer taxa representing potentially more specialized carnivore, insectivore, or herbivore forms were not included in our sampling. Third, the spatial discontinuity of stratigraphically younger (Eocene) and older (Cretaceous) mammal assemblages means that body size and ecomorphological shifts bracketing the Paleocene cannot currently be analyzed across the sampled basins alongside the dataset presented. These limitations should be considered when interpreting the findings reported in the study.

### Dental traits paralleled Paleocene global and regional environmental conditions

We posit that dental topographic trait variability in Paleocene eutherians in South China tracked global and regional climatic changes despite stasis in high-level taxonomic composition over the course of the first 10 Myr after the K-Pg transition (Figure 1). Dental height and sharpness variability were low in the beginning and end of the time interval, with a peak in the middle Paleocene. This pattern is observed both when dentitions are considered as pooled samples through time and particularly driven by the lower dentition (Figure 1—figure supplement 5; note that upper teeth display the opposite pattern). In contrast, elevated low-level taxonomic turnover of genera and species within the Paleocene indicates that cladogenetic shifts, rather than anagenetic adaptation, underpin this dental topographic evolution (Source data 1; Wang et al., 2007). These findings suggest that in addition to its impact on crown group eutherians, the K-Pg extinctions and subsequent climatic fluctuations played a role in filtering out archaic eutherians with lower speciation rates in favor of rapidly speciating taxa (Quintero et al., 2024).

By contrast, we found no support for significant shifts in dental topographic trait mean values from the early to the middle Paleocene for the majority of analytical iterations of clade and dental data partitions (Figure 1A, Figure 1—figure supplement 4; Table 1; Source data 7). However, in most analyses, we observed a significant shift in at least one dental topographic metric from the middle to the late Paleocene (Table 1; Source data 7). The larger-bodied Chinese endemic pantodont (an ungulate-like archaic placental group; ‘CEP’) mammals tend to increase dental complexity (OPCR) and curvature (DNE), whereas smaller, non-pantodonts (Chinese arctostylopids and anagalids; rodent-like archaic placental groups) in the dataset exhibited no significant dental trait shifts from the middle to late Paleocene (Figure 1—figure supplement 4; Source data 7). The transformation by CEPs in complexity and curvature indices relates to the capacity of teeth to resist wear and coincides with temperature and aridity increase toward the end-Paleocene thermal maximum event (Figures 1 and 2). Multiple geological and geochemical proxies suggest that paleoclimate in and around the Nanxiong Basin K-Pg section in South China reflects a latitudinally much broader global tropical zone during the Paleocene (Yin et al., 2023) relative to present-day Earth, as well as rapid shifts between more and less humid intervals during the first 10 Myr of the Cenozoic (Huang et al., 2024). Results from feldspar-quartz (F:Q) ratios, clay mineral composition analysis, diffuse reflectance spectroscopy, stable carbon isotope analysis, and total organic carbon analysis all support this regional paleoclimate profile (Huang et al., 2024; Wang et al., 2015). Local climate reconstructions for the latest Cretaceous indicate relatively warm and dry intervals, followed by warm and humid climates during the earlier Paleocene, then a return to less humid but still warm conditions in the later Paleocene (Wang et al., 2015). CEP dentitions tracked shifts in this paleoenvironmental progression.

**Table 1.** Pairwise t-test of dental topographic trait and disparity differences across adjacent time bins. Dental topographic trait differences are assessed across time intervals in all-data, Chinese endemic pantodont, and non-pantodont partitions. Dental trait disparity was estimated based on all principal component axes using the outputs of PCA. Tooth size variance differences were calculated from tooth area or square root of tooth area in all-data and no-outlier partitions to assess the effect of outliers on statistical significance (see Source data 6 for details). Bolded font indicates p-values<0.05.

All data	Early-middle Paleocene	Middle-late Paleocene
DNE	0.7753	**0.003**
OPCR	0.0902	**0.0001**
RFI	0.605	**0.0128**
Slope	0.643	0.287
PCA disparity	0.4562	**0.0001**
Tooth size	**<0.0001**	**<0.0001**
Chinese endemic pantodonts		
DNE	0.9264	**0.0002**
OPCR	0.17	**<0.0001**
RFI	1	1
Slope	1	1
Chinese arctostylopids and anagalids		
DNE	0.847	0.135
OPCR	0.47	0.18
RFI	0.2486	**0.0056**
Slope	0.642	0.269

The overall increase in dental complexity and curvature also coincided with an increase in drought-tolerant flora in South China (Figure 2) and specifically with paleoenvironmental reconstructions in the Nanxiong Basin (Ma et al., 2018). Palynological evidence suggests that in addition to a predominance of broad-leaved, deciduous plants mixed with a smaller percentage of conifers than in fossil localities further north (Hao et al., 2010), South China also recorded drought-resistant taxa such as the maidenhair fern *Pterisisporites* in the early Paleocene Shanghu formation (Xie et al., 2020) and again in late Paleocene samples (Ma et al., 2012). Expanded comparisons across time and space suggest fossil pollen taxa that are indicators of seasonal aridity increased from 20.3% of late Paleocene pollen samples to 34.3% of early Eocene pollen samples (Ma et al., 2012). These shifts appeared to have stabilized by the end of the Eocene, when taxonomically modern floras became established in Asia (Su et al., 2019). In addition to tracking Paleocene temperature trends (Figure 1), Asian eutherian dental topography (OPCR and DNE in particular) also mimicked trends in the marine realm, where global planktonic foraminifera records demonstrate a similar pattern in species richness curve, as well as with global δ^13^C (Keller et al., 2018; MacLeod et al., 2000) and south Pacific CO_2_ levels (Hollis et al., 2022). These broader associations underscore the inference that endemic Paleocene eutherians in South China comprised a dynamic assemblage shifting their dental morphology in step with regional and global environmental changes during the first 10 Myr after the K-Pg mass extinctions.

We detected a shift and expansion of eutherian dental morphospace in the late Paleocene (Figure 2). An overall shift toward increased dental topographic trait magnitudes in late Paleocene samples is driven mainly by CEPs (Table 1), despite the fact that they constitute a minority of the overall data (41% of teeth), as well as late Paleocene partition (21% of teeth). Additionally, dental metric disparity is significantly higher in the late Paleocene partition than in the two preceding time bins (Table 1). This pattern is driven by both increased dental curvature and complexity (DNE and OPCR; 78–100% support in per-tooth analyses, Figure 1—figure supplement 5) in larger-bodied CEPs and crown height (RFI) in smaller-bodied non-pantodonts, in addition to differences in disparity among the clades (Source data 7, Figure 1—figure supplement 5; bootstrapped variance for DNE, OPCR, and RFI are at least twice as large in non-pantodonts compared to CEPs, and bootstrapped range lengths returned similar patterns). This suggests that an expansion of dental disparity in the late Paleocene occurred across the size spectrum of endemic Asian eutherians. Over the same time interval examined, body (tooth) size disparity and mean were higher in the early Paleocene than in subsequent time intervals (Figure 1—figure supplement 6, Table 4; also supported by premolar 4 and upper molar partition analyses), indicating that substantial increases in the disparity of dental complexity, curvature, and height lagged behind tooth size during the Paleocene. Dog-sized CEPs such as *Bemalambda* reached sizes not seen in late Cretaceous mammals from China such as *Zhangolestes* and *Kryptobaatar*, which are shrew- to gopher-sized (Meng, 2014). This suggests a ‘brawn before bite’ pattern in endemic Asian eutherians, partially mirroring the endocranial and jaw functional morphology patterns identified in their North American and European counterparts (Benevento et al., 2019; Bertrand et al., 2022). These findings raise the possibility that an initial size-driven post-K-Pg recovery followed by ecomorphological radiation was a global phenomenon, even as regional tectonic events such as the initial collision of the Indian subcontinent with Asia and Deccan Traps volcanism influenced local mammal evolution (Hu et al., 2015; Ma et al., 2024).

### Topography-performance covariation underlies eutherian dental shifts

Within an overall pattern of increasing covariation between dental topographic traits and bite performance traits across the Paleocene time intervals (Figure 3; Table 2), topographic versus performance trait variability shifted both from early to middle and from middle to late Paleocene time bins (Figure 1—figure supplement 3 and Figure 1—figure supplement 4). Early Paleocene to middle Paleocene DTA-FEA intra- and inter-correlations remained stable (Figure 3B), but DTA-FEA inter-correlations strengthened while intra-DTA correlations weakened in the middle to late Paleocene transition (Figure 3C). This transition pattern is in part due to the divergence of shape-performance linkages in CEP versus non-pantodont eutherians, and is particularly driven by the upper and lower first molars (Figure 3—figure supplements 3–5). Dental topography variability tracked bite performance variability in non-pantodonts through time, peaking in the middle Paleocene (Figure 1—figure supplement 4D). By contrast, both intra- and inter-partition comparisons of topography and performance trends in CEPs showed low correlations through time (Figure 1—figure supplement 4G). The coexistence of distinct topography-performance relationships in each time and taxon partition while overall covariation between the two trait groups increases between time bins is consistent with form-function decoupling (Wainwright et al., 2005). Complex form-function linkages generally promote evolutionary redundancy and can enhance optimization of phenotypic traits when selective trade-offs are present (Pollock et al., 2025; Polly et al., 2016; Wainwright et al., 2005). The presence of functional redundancies underlies the high levels of dental topographic variability in Asian Paleocene eutherians. Alternatively, varying degrees of independence between the two performance traits and dental topographic traits analyzed could allow the two aspects of the dentition to evolve in a decoupled manner (Figure 3).

**Table 2.** Two-block partial least squares (2B-PLS) r coefficients from bootstrapped analyses. 1000 bootstrap samples of dental topographic analysis (DTA) and finite element analysis (FEA) values were taken from uniform distributions of trait uncertainty ranges, and 2B-PLS analysis conducted on each sample for early-middle Paleocene and middle-late Paleocene data partition pairings, respectively. All r values are statistically significantly different (p<0.05) between adjacent time intervals, based on a one-sample t-test of the distribution of 1000 p-values from the bootstrap 2B-PLS samples against p<0.05.

Taxon	Dentition	Early Paleocene	Middle Paleocene	Late Paleocene
1. All Taxa	All	0.40	0.48	0.52
2. Chinese endemic pantodonts	All	0.30	0.29	0.43
	Premolar	0.69	0.57	0.51
	Molar	0.15	0.70	0.65
3. Non-pantodont mammals	All	0.48	0.53	0.49
	Premolar	0.50	0.52	0.55
	Molar	0.45	0.40	0.46

### Insights on evolutionary preconditions for the post-K-Pg eutherian radiation

Ting et al., 2011, described an in situ faunal turnover event in southern China between the early and middle Paleocene and a gentle decrease in endemic taxa. Such a faunal change did not amount to shifting dental topographic mean values, although it is correlated with increased variability in all topographic traits (Figure 1). Similar inferences of ecological stasis were made by Clyde et al., 2008. The replacement of some archaic taxa with more cosmopolitan ones is consistent with an evolutionary ‘training ground’ phenomenon where taxa preadapted to local environmental conditions were at a selective advantage to disperse into new geographic regions when global climate changed (Deng et al., 2011). Hooker, 2000, makes the observation that late Paleocene North American and European mammal assemblages responded to the late Paleocene thermal maximum by increasing browsing herbivory and terrestrial taxa; this increase is at the expense of arboreal taxa in the European samples analyzed in that study. The observed DTA shifts in our dataset are consistent with this trend (Figure 2). In the final Paleocene time bin, immediately prior to the faunal turnover that coincided with the Holarctic mammalian dispersal event and the PETM (Paleocene-Eocene Thermal Maximum), increased seasonal aridity in southern China (based on pollen composition) correlated with increased disparity and maxima of dental topographic metrics (Figure 2).

The majority of research into post-K-Pg mammal recovery comes from North American sites, which represent ~80% of known terrestrial K-Pg boundary sections (Vajda and Bercovici, 2014). By contrast, Asian K-Pg sites represent only ~3% of K-Pg boundary sections. There are no Paleocene fossil mammal assemblages known from the Indian subcontinent (Ni et al., 2020). Europe is similarly challenging in terms of both the paucity of well-dated K-Pg sites and correlated or thick stratigraphic sections that permit research into post-K-Pg dynamics (Csiki-Sava et al., 2015). Additionally, the oldest well-sampled Cenozoic mammal fauna in Europe is late early to middle Paleocene in age. This absence of earliest Paleocene data renders the study of mammalian evolutionary dynamics from the K-Pg to end-Paleocene hyperthermal event in Europe difficult (De Bast and Smith, 2017). Thus, no dental form-function comparisons are possible between the Asian and European post-K-Pg mammal recovery patterns for the entire Paleocene time interval.

Bayesian analyses of Paleogene mammal faunas suggest that a zoogeographic barrier between the Arabian Peninsula and the rest of Asia was present and prevented faunal interchange until the end-Oligocene (Ni et al., 2020). Thus, the corridors of dispersal and interchange for the southern Chinese Paleocene mammals at the end-Paleocene were constrained to the north, toward the Mongolian Plateau, or to the south toward India. The absence of Paleocene Indian fossil mammals prevents a characterization of the extent to which the ecomorphological patterns described in this study also apply more broadly to fossil mammals further to the south. Based on the establishment of a land connection between Asia and India during the Paleogene, similarity in ecomorphological patterns might be expected (Hu et al., 2015). By contrast, the well-known Paleocene-Eocene stratigraphic sequence and fossil record on the Mongolian Plateau clearly document the northward expansion of early Paleocene southern Asian taxa and the increased faunal similarity with other continents (Wang et al., 2010) during time intervals immediately following those analyzed in this study.

Absence of latitudinal gradients in Paleocene mammal taxonomic richness despite modern-level temperature gradients across western interior North America suggests potential ecological instability in that region around the time of the mammalian dispersal to North America (Rose et al., 2011). However, other analyses suggest that the arrival of Eurasian immigrants to North America at the PETM did not drastically alter functional diversity in the local communities (Fraser and Lyons, 2020). Establishment of latitudinal floral gradients and the high mammal endemism in east Asia during the same time period (Qiu and Li, 2005) may have allowed ecological stability to establish earlier in Asia compared to North America. The stability of a large landmass in Asia for much of the Mesozoic and Cenozoic likely also contributed to the development of climate-adapted faunas in south Asia leading up to the PETM and expansion of suitable warm habitats for the southern taxa (Beard, 1998). Data presented here suggest that adaptations of endemic east Asian mammals to global and regional environmental changes in the critical 10 Myr period immediately following the K-Pg extinctions can be understood as priming the evolutionary pump for the origin, radiation, and dispersal of modern mammal orders.

### South Asia as a Paleogene ‘Garden of Eden’ for eutherian mammals?

Among the most consequential implications of accurately interpreting post-K-Pg mammalian recovery dynamics in Asia is the ability to reconstruct the evolutionary conditions during the biogeographic origination of modern placental orders. Current knowledge and the overwhelming majority of data on the first post-dinosaur mammal ecological communities are centered on North American localities (Rose, 1981; Wing et al., 1995) and, secondarily, on European records (Hooker, 2000). Yet, the fossil records of those continents pinpoint a high turnover of mammal faunas at the Paleocene-Eocene boundary, driven by the Cretaceous/Paleocene origination of major modern mammal clades elsewhere and subsequent dispersal of those lineages to the European and North American continents, respectively. At least five living orders (Primates, Rodentia, Lagomorpha, Perissodactyla, Eulipotyphla), representing over half of mammalian species richness today, trace their evolutionary origins to Asia (Beard, 1998; Gingerich, 2010; Lopatin, 2006; Maas et al., 1988). There is also mounting evidence that other organisms, including fish and plant lineages, followed a similar biogeographic pattern (Culver and Rawson, 2006).

The abrupt appearance of early representatives of modern mammal lineages in North America has been formally operationalized by the ‘East of Eden’ model, wherein Asia is the ‘Garden of Eden’ and a biodiversity pump of living orders of eutherian mammals (Beard, 2002) and other organisms. Biogeographic modeling analysis of modern mammalian diversity also strongly identifies tropical south Asia, including the geographic regions occupied today by southern China, India, and southeast Asian countries, as the cradle of mammalian diversification since the Paleocene (Feijó et al., 2022). As geographically proximate faunal assemblages to the epicenter of the ‘Garden of Eden’ hypothesis, the endemic Paleocene eutherians of the Asian paleotropics analyzed in this study exhibited a high degree of dental topographic variability while strengthening topography-performance covariation during a period of biogeographic isolation and regional and global climate changes. Such flexibility in dental form-function linkage permits ‘mix and match’ trait combinations rather than evolutionary transformation as a single unit, potentially enhancing the evolvability of feeding ecological traits as new environmental conditions arose (Goswami et al., 2015). This finding favors the scenario whereby many living orders of placental mammals were borne out of phenotypically and functionally plastic ancestral assemblages, including those that lived in South China, during the Paleocene (McKenna et al., 1989).

We further hypothesize from these findings that episodes of fluctuating global warming during the first 10 Myr of the Cenozoic promoted the evolution of ‘all-purpose’ mammalian dentitions rather than those with specialized functions (Figure 3). This prolonged dental topographic variability tracks extended post-K-Pg floral recovery times (Ma et al., 2012; Peppe, 2010) and suggests a ‘brawn before brains’ mode of placental mammal evolution may in part represent a series of correlated evolutionary shifts in sync with the steadily increasing complexity of Paleocene primary producers (Su et al., 2019; Wilson, 2013). That a global signal would be detected in the splendidly isolated South Asian Paleocene assemblage is notable and indicates that the effects of climate forcing on post-K-Pg mammal recovery may be ubiquitous, as has been predicted for Earth’s ongoing rapid environmental shifts (Scheffers et al., 2016). In response, Paleocene mammal clades in South China were relatively larger early on and increased covariation between dental topography and bite performance later, all the while maintaining high levels of variability in dental complexity and convexity (Figure 1). These preconditions may have set the stage for the subsequent taxonomic turnover between archaic and crown lineages during the Paleogene to Neogene modernization of mammal communities (Bowen et al., 2002; Ting et al., 2011). As a primary interface against shifting food resources and the environment, the eutherian dentition was poised to play an outsized role in the explosive diversification following the K-Pg extinctions, with the masticatory complex having already completed the macroevolutionary transition to a mechanically stiff but phenotypically flexible jaw during the Mesozoic Era (Schultz, 2025; Tseng et al., 2023). The end-Cretaceous extinctions and the climatic volatility that followed then set in motion the ecological release that enabled mammals to explore dental form-function to a far fuller extent in 66 Myr of the Cenozoic Era than during the preceding 150 Myr under the reign of dinosaurs. This new hypothesis of ‘brawn before bite’ in tooth size, topographic, and performance evolution further refines the placental success story.

## Materials and methods

### Field locality information

The extensive development and conversion of outcrops in the Nanxiong Basin into agricultural fields has created a ‘race against time’ to document and study this critical area to understand placental evolution. Clyde et al., 2010, estimated that 20 out of 54 early and middle Paleocene fossil sites in the Nanxiong Basin have already been destroyed by infrastructural and housing developments since their original discovery nearly three quarters of a century prior. Our re-survey of the key fossil sites during a trip there in 2023 suggests that the majority of the sites, except for the K-Pg boundary locality, which is protected by municipal historic landmark designation, are now inaccessible or completely obliterated. This reality means that the mammalian fossil samples analyzed in this study offer an ever more important and rare window into earliest mammal life during the ‘Age of Mammals’. Our analyses are based on the most complete earlier Paleocene materials currently recovered from the Asian continent.

Isotope analyses show that average δ^13^C values returned to pre K-Pg levels from a short-duration 2 ppm (part per mil) decrease about 1 Myr into the Paleocene in the Nanxiong Basin, congruent with global patterns of rapid mammal taxonomic recovery (Clyde et al., 2010). Although the majority of Paleocene fossiliferous localities are within the classic ‘red beds’ of South China, interbedding with limestone-like layers may indicate localized cycles of dry and humid intervals (Xie et al., 2019). Fossil pollen in the Nanxiong Basin indicates warm climates throughout the Paleocene time interval; however, a notable shift in higher percentages of ferns and gymnosperms and lower percentage of angiosperms is observed in the late Paleocene (Zhang et al., 2021), indicating local recovery of gymnosperms and ferns (Table 3).

**Table 3.** Relative percentages of fossil pollen found in the Nanxiong Basin based on Zhang et al., 2021.

	E. Paleocene	M. Paleocene	L. Paleocene
Angiosperms	75–88%	84%	50–65%
Ferns	11–20%	11%	25–33%
Gymnosperms	1–5%	5%	10–18%

The antipodal location of the Nanxiong Basin from the Yucatan Peninsula makes the geochemical identification of a K-Pg boundary layer difficult. Arguments for the precise location of the K-Pg boundary layer have been made on the basis of iridium enrichment in dinosaur egg shells (Zhao et al., 2002) and with total mercury content that correlates with known timing of Deccan Traps volcanism (Zhao et al., 2021). Regardless of the precise stratigraphic level of the K-Pg boundary in the Nanxiong Basin, the specimens we included in our analyses have all been collected in higher stratigraphic levels that are biostratigraphically unambiguous as Paleocene sequences (Zhang et al., 2021). Planned fieldwork at and around the putative K-Pg boundary sections will sample micromammal fossils toward the objective of constructing a refined biostratigraphic framework for high-resolution analysis of the first million years of post-K-Pg mammal recovery. At this time, high-resolution data are not available to test the precise rate at which Paleocene Asian mammal taxonomic disparity and size disparity recovered/stabilized (Table 4).

**Table 4.** Sample size, disparity, and mean tooth size by tooth position. Figure 1 Mean disparity difference is measured by pairwise variance tests (var.test); mean tooth size difference is measured by pairwise t-tests (t.test). p-Values≤0.05 are underlined. Overall disparity trends are also observed in premolar and upper molar data partitions, whereas overall tooth size trends are observed mainly in the lower premolar 4 data partition. Decrease in mean tooth size is most consistently observed across multiple tooth partitions for the early Paleocene to late Paleocene comparison.

Measure	Trait	Statistic	All	m/1	m/2	m/3	M1/	M2/	M3/	p/4	P3/	P4/
Sample size	Early Paleocene (EP)		79	10	11	8	10	10	8	7	8	7
	Middle Paleocene (MP)		42	5	6	6	4	5	4	4	4	4
	Late Paleocene (LP)		52	5	5	5	8	8	4	6	3	8
Mean disparity	Area	var.test EP-MP	0.00	0.44	0.81	0.78	0.02	0.97	0.01	0.00	0.54	0.04
		var.test MP-LP	0.03	0.00	0.06	0.92	0.14	0.00	0.04	0.08	0.05	0.24
	Sqrt area	var.test EP-MP	0.06	0.59	0.56	0.59	0.13	0.74	0.14	0.04	0.44	0.12
		var.test MP-LP	0.16	0.02	0.11	0.72	0.28	0.06	0.07	0.30	0.17	0.72
Mean size	Area	t.test EP-MP	0.00	0.18	1.00	1.00	0.75	0.91	0.06	0.04	0.86	0.53
		t.test MP-LP	0.01	0.18	1.00	1.00	0.75	0.18	0.40	0.04	0.34	0.03
		t.test EP-LP	0.00	0.01	1.00	1.00	0.75	0.02	0.01	0.00	0.34	0.00
	Sqrt area	t.test EP-MP	0.00	0.18	1.00	1.00	0.75	0.91	0.06	0.04	0.86	0.53
		t.test MP-LP	0.01	0.18	1.00	1.00	0.75	0.18	0.40	0.04	0.34	0.03
		t.test EP-LP	0.00	0.01	1.00	1.00	0.75	0.02	0.01	0.00	0.34	0.00

### Data sampling

We focused sampling on three clades: Chinese endemic Pantodonta, Chinese Arctostylopida, and Anagalida. Additional data were collected on other clades (e.g. Chinese Tillodontia) opportunistically when well-preserved specimens were available (Source data 1). These three main clades together represent >50% of the species found in Paleocene faunas across China (Wang et al., 2007). They also individually represent the most diverse clades across the three Asian Land Mammal Ages of the Paleocene: Shanghuan, Nongshanian, and Gashatan. By contrast, these clades are reduced to <25% of species diversity in Eocene assemblages in China, finally disappearing altogether in the late Oligocene. Therefore, we take the three clades to be representative of eutherian assemblage dynamics during the Paleocene time interval in China.

Historically, Paleocene fossil mammal faunas in China have been defined based on biostratigraphic criteria and supplemented by magnetostratigraphic correlations where available. Here, we follow the assessment of Speijer et al., 2020, in generally correlating the Shanghuan Asian Land Mammal Age (ALMA) with the Puercan and Torrejonian North American Land Mammal Ages (NALMAs), the Nongshanian ALMA with early to middle Tiffanian NALMAs, and the Gashantan ALMA with late Tiffanian and Clarkforkian NALMAs, respectively (see Wang et al., 2007, for an alternative interpretation). Additionally, the Shanghuan and Nongshanian boundary was interpreted by Clyde et al., 2010, to be at the top of Chron C27N, which is dated at 60.920 Myr ago in the Geomagnetic Polarity Time Scale (Cande and Kent, 1995) but indicated as closer to ~62.3 Myr ago in Speijer et al., 2020. Similarly, the Nongshanian-Gashatan boundary has been variably defined at 59.24 Myr ago (Speijer et al., 2020) or the base of Chron C26N, which is 57.911 Myr ago (Cande and Kent, 1995). In contrast, the end of the Gashatan coincides with the Paleocene-Eocene boundary at 56 Myr ago and has been consistently defined as such (Speijer et al., 2020). Given these existing uncertainties, we use the terms ‘early Paleocene’, ‘middle Paleocene’, and ‘late Paleocene’ to refer generally to the Shanghuan, Nongshanian, and Gashatan ALMAs, respectively, in this study.

We analyzed 200 individual teeth from 48 specimens, representing 37 species (Source data 1 and 2). The teeth represent 2 upper first premolars, 4 upper second premolars, 15 upper third premolars, 19 upper fourth premolars, 22 upper first molars, 23 upper second molars, 16 upper third molars, 3 lower first premolars, 5 lower second premolars, 13 lower third premolars, 17 lower fourth premolars, 20 lower first molars, 22 lower second molars, and 19 lower third molars. These tooth positions were selected from a broader examination of ~300 individual teeth from 72 specimens. We vetted the specimens and excluded 99 tooth positions (~33% of teeth initially chosen for possible inclusion) from our analyses because they either (1) were partially or completely broken at the crown, (2) were in an advanced stage of attritional wear where no cusps could be identified, or (3) possessed a combination of the two aforementioned conditions. The assigned geologic age of the studied specimens spans the entire Paleocene, binned into early (n=91 teeth), middle (n=46 teeth), and late Paleocene (n=63 teeth) data partitions. To maximize sample size while minimizing disturbance to more delicate specimens, a combination of original specimens and previously produced high-fidelity specimen casts were used in our sampling of dental crown morphology.

Given the rarity of Paleocene fossil material from China, we combined data from different tooth positions into three pooled samples, one for each of the time intervals examined (early, middle, late Paleocene). We treated the pooled samples as representative of the range of dental topographic features and bite performance traits available to the eutherian taxa under study. In this way, the variance estimates are interpreted as measures of the morphological and performance heterogeneity present in each time interval dataset. To further tease out the possibility of specific tooth positions driving the overall trends observed in the pooled samples, we also performed the DTA, FEA, DTA-FEA correlation, and tooth size through-time analyses using per-tooth data partitions.

### Tooth shape estimates using DTA

We digitized dental crown morphology via microCT using either a GE v|tome|x m 300/180 kV microcomputed-tomography system (GE Measurement & Control Solutions, Wuntsdorf, Germany), housed at the Institute of Vertebrate Paleontology and Paleoanthropology (IVPP), Chinese Academy of Sciences (CAS), or a GE Phoenix Nanotom M in the Functional Anatomy and Vertebrate Evolution Laboratory, University of California, Berkeley. Projection images (1000–1500 images depending on specimen size) were acquired with an isotropic voxel size of 10–40 μm, at an energy range of 120–150 kV, current of 100–150 μA. Additionally, surface 3D scans (generated using IVPP’s Artec Space Spider 3D scanner, with a precision of 0.05 mm and resolution of 0.1 mm) were used for larger specimens to efficiently obtain surface morphology data. The enamel caps (the crown portion of each tooth above the enamel-dentine junction) were extracted from the specimen models using Geomagic Wrap v2021; all crown surface holes (generated from scanning imperfections or digitally removed sediment) were patched, the models were remeshed with triangles that have aspect ratios (base:height or edge-edge) of <10 and decimated to ~10,000 triangles. Finally, we standardized the spatial orientation of all tooth crowns before exporting them as .ply files for DTA. Individual tooth crown (enamel cap) models have the Z axis normal to the occlusal plane, the X axis parallel to the mesial-distal axis, and the Y axis parallel to the labial-lingual axis.

Exported meshes were further vetted and prepared for DTA within the R programming environment. We used functions implemented in the *molaR* R package (Pampush et al., 2016) for all of the steps described next. First, all .ply files are further cleaned using ‘molarR_Clean’ to remove floating points/vertices and any triangular faces with zero area. Then, the batch function ‘molarR_Batch’ was used to calculate four dental topographic metrics: DNE, OPCR, Slope, and RFI.

DNE is a measure of occlusal sharpness, using a quantification of surface energy in tooth crowns relative to a gently curving or flat mesh surface. Steep, high, and/or shearing cusps tend to produce higher DNE values in DTA, whereas bulbous cusps tend to produce lower DNE values (Bunn et al., 2011; Waldman et al., 2023). DNE has been used to successfully distinguish folivores, omnivores, and frugivores in euarchontan (primates, colugos, tree shrews) mammals (Berthaume et al., 2020; Bunn et al., 2011).

OPCR is a measure of tooth crown complexity, using quantification of distinct patches on the crown face that possess unique orientations. Teeth with a larger number of cusps and crenulations tend to produce higher OPCR values, whereas teeth with fewer cusps and simpler ridges tend to produce lower OPCR values. This metric has been used effectively to distinguish convergently evolved carnivore versus herbivore species across multiple mammalian orders (Evans et al., 2007). DeMers and Hunter, 2024, suggest that OPCR does not currently provide a reliable approximation for the degree of adaptation to herbivory, and that any interpretation of ecomorphological differences should be made within a taxon-specific context. Given the narrow set of clades targeted in our dataset, we interpret increasing OPCR as an indication of increased functional capability to process vegetation. A related measure, slope, quantifies the mean tilt of the tooth surface relative to the occlusal plane. Teeth with lower and more gently curving cusps tend to have lower slopes, whereas teeth with sharp and/or abruptly ridged crests tend to have higher slopes.

RFI is a measure of the relative height and complexity of the tooth crown, using the values of the 3D surface and 2D ‘footprint’ areas of the tooth crown. RFI has been shown to be effective in distinguishing frugivores from insect and leaf specialists in euarchontan mammals (Bunn et al., 2011). Furthermore, RFI is also able to distinguish carnivoran species at different trophic levels and dietary breadths (Waldman et al., 2023).

Overall, we use these DTA traits as indicators of ecomorphological capacity, but do not link them explicitly to dietary categories. The craniodental morphology of archaic placental clades in general has not been demonstrated to share the same structure-function linkages as crown mammals, so aforementioned linkages between DTA and dietary ecology from studies of extant species only serve as evidence that DTA is a potentially useful ecomorphological proxy. We do not directly apply those DTA-diet relationships to the Paleocene fossil eutherian dataset.

All tooth crown models are provided as ply files and deposited in FigShare (10.6084/m9.figshare.28611854).

### Tooth performance estimates using FEA

The common inference that dental morphology can reflect dietary adaptations makes an underlying connection between the two traits through biomechanical performance; the mechanical performance imbued by a particular morphological configuration accomplishes one or more food acquisition and/or processing tasks. Such mechanical performance links can be tested using experimental and simulation approaches on both theoretical grounds and actual tooth shapes (Crofts et al., 2020). Here, we applied a simulation approach to estimate two general performance traits commonly interpreted for mammalian tribosphenic teeth: puncturing/compressing and shearing/grinding (Crompton and Hiiemae, 1969). The ancestral therian tribosphenic stroke, or the chewing movement underlying mammals with unfused mandibular symphysis and tribosphenic molars, has been reconstructed to involve significant components of (1) long-axis rotation in each hemimandible and (2) mortar-and-pestle grinding between upper and lower cusps (Bhullar et al., 2019). These movements are associated with the ‘lock-and-key’ occlusion of a stereotypical mammalian tribosphenic dentition, which exhibit tall crest-like cusps in the trigonid and lower basin-like valleys in the talonid of lower molars that articulate with their corresponding upper teeth (Figure 1—figure supplement 3).

We used FEA to estimate the work efficiency of different tooth crowns subjected to compressive/puncturing and shearing/grinding forces, the two major masticatory actions of tribosphenic teeth (Crompton and Hiiemae, 1969; Simpson, 1936; Yamanaka et al., 2024). Adequate compressive and puncturing forces, applied through individual or combined action of cusps, are needed to overcome the fracture strength of brittle and tough food such as seeds and invertebrate exoskeletons in order for individuals to access softer and/or more nutritious tissues within. Sufficient shearing and grinding forces applied through surfaces or cusp edges are necessary to further break down food boluses into smaller pieces to aid in the digestive process. The combination of these two major functions of tribosphenic teeth is thought to be a foundational adaptation that enabled the mammalian radiation (Bhullar et al., 2019; Crompton and Hiiemae, 1969; Lucas and van Casteren, 2015). We focused on the dental enamel portion of the tooth crown in our biting simulations because it is the most mineralized vertebrate tissue and typically the best-preserved component of fossil teeth. The fracture mechanics of mammalian dental enamel is thought to be intimately related to dietary function (van Casteren and Crofts, 2019), including the evolution of different enamel thicknesses in different feeding ecomorphologies (Lucas et al., 2008). It is important to note that because the dental data available for this study included not only CT scans, but also surface scans and specimen casts, enamel thickness was not quantifiable for most specimens in our dataset (see below for a description of the enamel thickness scaling procedure applied in the simulations).

The two masticatory scenarios tested, compressing/puncturing versus shearing/grinding, have previously been shown to be broadly connected to tooth topology. Taller cusps (approximated in our study by RFI and partially by Slope) and more convex crowns (approximated in our study by DNE) tend to exhibit higher strain in finite element models of hypothetical tooth shapes (Crofts, 2015). However, there is by no means a consensus on the presence of a tight form-function linkage across all tooth types and/or clades. Whereas a dental topography to food mechanical property linkage can be detected in some extant carnivores (Pérez-Ramos et al., 2020; Pollock et al., 2022), bats (Villalobos‐Chaves and Santana, 2022), and primate-like tooth models (Sender and Strait, 2023), a form-function relationship between dental topography and tooth/food mechanical properties is absent in other model systems (Berthaume, 2016; Berthaume et al., 2020). Therefore, we incorporated biomechanical simulations as an explicit test of the mechanical performance associations implied by the DTA-flora correlations established in the dental topography and paleoenvironmental portion of our analyses. The main question addressed by these simulations is not necessarily whether a form-function linkage exists in the Paleocene eutherian dataset, but whether there is a consistent relationship between DTA and FEA traits across the three time bins of the Paleocene. If such consistent linkages are observed, it would suggest a stasis in dental topography and compressive/shearing performance through the first 10 Myr of the Cenozoic in our dataset; alternatively, any significant changes in the topography-performance relationship would indicate substantial evolutionary change through the same time period.

We imported the same tooth crown models generated for DTA into the Strand7 finite element software (Strand7 Pty Ltd, Australia) to create finite element models of the individual teeth. The tooth meshes are composed of 2D, three-noded triangular elements ranging between 9000 and 10,000 elements, with assigned thickness parameter (see below) so they behave similarly to single-layer 3D elements. A convergence test was done on two of the specimens (IVPP V5228, V5231) and indicated that the <10,000 three-noded triangular plate elements (tri3) returned simulation output values with less than 10% deviation from higher resolution meshes of the same models (Figure 1—figure supplement 2). Therefore, we deemed the default resolution of the tooth meshes used in DTA to be sufficient for finite element simulations. Furthermore, because the dataset contained digital models built from both CT and surface scan data, not all specimens have enamel thickness information. Although increased enamel thickness has been associated with durophagy in mammals, there appears to be taxon-specific patterns of enamel thickness to tooth size and dietary ecology, in addition to extensive variation in intra-tooth enamel thickness distributions (Dumont, 1995; Shellis et al., 1998; Ungar, 2015). In absence of information on enamel thickness differences among the taxa studied, we standardized the thickness of the tooth crown models isometrically to be 10% of the total surface area of a given tooth model. As such, the simulation results should be treated as enamel cap shape-derived performance traits rather than those based on a fully parameterized enamel model incorporating localized thickness and inter-specific allometric differences.

We scaled applied forces on the teeth, simulating compressive or shearing loads, to be a value equivalent to total model surface area (but in units of Newtons instead of mm^2^). Compressive loads were represented on each tooth by a single nodal force vector directed toward the base of the crown along the height axis of each tooth, on the tallest cusp or cuspid (which is typically the protocone for upper molars and protoconid for lower molars, or the main cusp in premolars). This configuration simulated a compressive load directed into the tallest cusp (toward the root) on a given tooth from a food item (Figure 1—figure supplement 1), as reconstructed for the mammalian tribosphenic bite during a crushing or puncturing movement (Bhullar et al., 2019; Crompton and Hiiemae, 1969). Shearing loads were represented on each tooth by two nodal force vectors, one on each of the two tallest cusps or cuspids; the two nodal forces are of equal magnitude but opposing directions, perpendicular to the long axis of the tooth and in the occlusal plane. This configuration portrays a shearing motion along the short axis of the tooth (Figure 1—figure supplement 3) and simulates tooth-to-tooth and tooth-to-food contact during the ‘mortar and pestle’ grinding rotation movement reconstructed for the ancestral mammal tribosphenic bite (Bhullar et al., 2019). This force magnitude standardization procedure effectively generates identical force to area ratios across the models, ensuring that the magnitudes of mechanical stress placed on the model are the same across models of different sizes; furthermore, we adjusted the strain energy values collected for each tooth model simulation using the volume and input force ratio-based correction equation provided in Dumont et al., 2009. Given our objective to assess the association between relative performance traits and DTA metrics, with the latter being size-free variables, only the relative magnitudes of bite performance, rather than absolute values, were collected from the performance analyses.

After each tooth crown model was defined using the criteria outlined above, homogenous material properties were assigned. All models were defined as plate models (with thickness scaled to surface area) with a Young’s (elastic) modulus of 80 GPa (He et al., 2006) and Poisson ratio of 0.3. Next, each tooth was constrained from translation and rotation using four nodal constraints distributed in the four corners of each tooth, respectively (Figure 1—figure supplement 3).

All bite scenarios were solved using the linear static solver implemented in Strand7. A total number of 400 analyses were performed (one compressive bite and one shearing bite simulation for each of the 200 tooth specimens in the dataset). We extracted two traits from the models: compressive bite strain energy and shear bite strain energy. Strain energy is defined as the area under a stress-strain curve of an object under load; operationally, it measures the amount of work done by the load to deform the object (analogous to experimental work-to-fracture measurements) (Conith et al., 2016). An object that is more resistant to deformation would have lower strain energy than an object that easily deforms under load. The use of work-to-fracture measures to assess tooth performance is consistent with the understanding of mammalian tooth enamel as a fracture-prone, yet fracture-resistant, biological tissue (Lee et al., 2011). In such a framework, fracture resistance is expected to be directly related to the amount of force an individual is able to exert through the tooth crown during mastication, and by extension the hardest or toughest food item that can be processed without catastrophic damage to the tooth crown itself. Fracture resistance (as approximated by strain energy in this study) is also likely to be a stronger target of selection in mammals compared to those of other toothed vertebrates, given the former’s diphyodonty (having only two sets of teeth instead of continuous dental replacement) and thus necessity to prolong usable tooth lifespan (Ungar, 2015). Therefore, we used strain energy values under compressive and shear bite simulation scenarios as a proxy for the effectiveness of each tooth crown model at resisting deformation from the respective biting forces. A tooth that is more resistant (i.e. has lower strain energy values) to compressive forces would be able to more effectively crush/puncture harder food items, and a tooth more resistant to shear forces would be able to cut/grind tougher food items. We modify Irschick et al., 2008, definition of whole-organism performance and define bite performance in the context of this study as the capacity of individual tooth models to resist simulated compressive and shearing forces, which represent ecologically relevant factors that influence masticatory efficacy.

All finite element models are provided as NASTRAN/text files and deposited in FigShare (10.6084/m9.figshare.28611854).

### Sensitivity validation of original versus cast specimen models

The tooth dataset developed in this study contains a mixture of CT scans of original and casts of fossil specimens. In order to assess the potential discrepancies in DTA and FEA trait values collected from cast versus original specimen-derived models, either due to phenotypic details not captured in cast specimens or deterioration of epoxy or resin-based casts over time, we randomly sampled two specimens for which both original and cast-based models were analyzed. Results from DTA and FEA of the CT-derived models of lower first molars from IVPP V5228 (*A. pactus*) and V5231 (*A. tenuis*) were compared to those conducted on digital models built from CT scans of casts of those specimens.

We used the ‘n-point’ and ‘global’ alignment functions in Geomagic Wrap to first align the models using arbitrary homologous landmarks visually identified on both tooth models and then the automatic global alignment function to maximize overlap between the two models in 3D coordinate space. The ‘deviation’ function in Geomagic Wrap was then used to calculate summary statistics for linear deviations orthogonal to the surface of the original model. The V5228 Lm1 model showed a maximum deviation of 0.58 mm (5.8% of crown height) and average deviation of 0.04–0.09 mm (<1% of crown height), with a standard deviation of 0.11 mm (1% of crown height). The V5231 Lm1 model showed a maximum deviation of 0.38 mm (6% of crown height) and average of 0.03–0.04 mm (<1% of crown height), with a standard deviation of 0.06 mm (1% of crown height) (Figure 1—figure supplement 3).

### Quantification of uncertainty ranges for bootstrap sampling

We then subjected the cast-based models to DTA and FEA. In both cases, the DTA values between cast and specimen models are the most different for DNE (30–40% difference), followed by OPCR (18–32% difference), then RFI (2–13% difference), and finally Slope (1–5% difference) (Source data 4). Also in both cases, adjusted Compressive SE values differed by 14–41% and adjusted shear values differed by 20% (Source data 3). Based on these specimen-cast validation tests, we set a conservative ±40% uncertainty range for all DTA and FEA values obtained from all cast-based tooth models. DTA and FEA trait mean and variance estimates used for downstream statistical analyses were then calculated using a bootstrap sampling scheme where 1000 replicates of the DTA and FEA datasets were passed through the statistical tests (see Quantification and statistical analysis section, below). Overall, the early and middle Paleocene time bins contain around 50% cast data, whereas the late Paleocene time bin contains 66% cast data. If data uncertainty between original and specimen models presents a major signal, we would expect the late Paleocene time interval to always show higher variance given an abundance of cast data in that time bin. We did not observe any consistent trends of high variance in the late Paleocene in the 1000 bootstrap samples, suggesting that the results are not significantly biased by data quality differences between cast and original specimen-derived models (Figure 1—figure supplement 4).

### Time bin duration correction and tooth position ratios

According to Ni et al., 2020, the Shanghuan ALMA faunas in Guangdong and Anhui range from 66 to 61.6 Ma (or 4.4 Myr in duration), the Nongshanian ALMA faunas in Guangdong, Jiangxi, and Anhui range from 61.6 to 59.2 Ma (or 2.4 Myr in duration), and the Gashatan ALMA faunas in Anhui range from 59.2 to 56 Ma (or 3.2 Myr in duration). Given the different durations represented in each of the three time bins used in our analyses, we corrected all variance estimates by dividing the variance calculated in each of the bootstrapped samples by the time duration of the respective time bin. In this manner, the variance values reported in the study represent per-million-year values.

We used pie chart analysis to verify that the proportions of tooth positions in each time bin are not substantially different from each other (Figure 1—figure supplement 1). However, we caution that time bin comparisons of individual tooth positions are unlikely to be statistically robust because of small sample sizes. We only use aggregates of dental positions (all teeth, molars, or premolars) in our data partition analyses and interpretation.

### Quantification and statistical analysis

We used the following features of the dental topography and performance data as the basis for assessing faunal assemblage trait shifts through the Paleocene: trait mean, variance, trait-to-trait correlation, and partition-to-partition correlation. We used ANOVA (analysis of variance) and pairwise t-tests to compare trait means, F-tests to compare trait variance, linear regression analysis and Kendall’s τ to compare trait-to-trait correlation, and 2B-PLS analysis to compare partition-to-partition correlation. There are a total of six traits forming two main data partitions: topographic partition (DNE, OPCR, Slope, RFI) and performance partition (Compressive SE and Shear SE). To assess the sensitivity of the results to subsets of the data, all of the trait comparisons were done iteratively using the total dataset, by time bin (early, middle, late Paleocene), taxon (CEP versus non-pantodonts), and/or by dental position (all teeth, molar teeth, premolar teeth). All statistical tests were performed on 1000 bootstrap resampled datasets that were parameterized using results of sensitivity tests on original versus cast specimen models, finite element mesh convergence tests, and time bin duration comparisons (see previous section). In addition, we quantified tooth size using two measures: total 2D surface area and square root of surface area. The R script for all statistical analyses and plots is included in Source data 9.

### Statistical resampling and tests

We used bootstrap resampled variance of dental topographic metrics and dental performance variables as a measure of tooth form-function disparity. 1000 replicates of the 200-specimen DTA-FEA trait datasets were generated by sampling each DTA and FEA trait value from a uniform distribution defined using a ±40% range of the original model-derived values. The bootstrap resampling was done with replacement, so it was possible for a given DTA/FEA trait value in a sampled tooth model to be repeated in a different replicate, but very unlikely for entire datasets to share similar values with another replicate. We used this resampling scheme to account for the uncertainty in trait estimates introduced by the totality of modeling, specimen preservation, cast versus original, and other potential sources of uncertainty in trait values. For each replicate, variance was calculated for each metric, time interval, for CEP versus non-pantodont, and molar versus premolar data partitions. To assess shifts through time, statistical differences between the variance of each dental topographic metric sample in adjacent time intervals (A, early Paleocene; B, middle Paleocene; C, late Paleocene) were evaluated using F- tests. A null hypothesis of a variance ratio of 1 between pairwise comparisons was tested using the var.test() function in R (R Development Core Team, 2025). The outputs of the variance tests on a given bootstrap sample were then compiled for all 1000 replicates, and the overall variance mean and variance test output used as the statistical basis for detecting significant differences between time, taxon, and tooth data partitions. Unless indicated otherwise, all tests described below were also done using the 1000 bootstrap samples.

We evaluated differences in central tendency (mean value) of dental topographic metrics and dental performance variables through time using ANOVA and pairwise t-tests. Each dental topographic metric was tested against the three time intervals defined above, using the aov() function in R. For statistically significant (at the p<0.05 level) results, we additionally evaluated the pairwise intervals that contribute significant differences in mean dental topographic values. We assessed pairwise differences using pairwise t-tests implemented with a Holm correction for multiple comparisons. The t-test was conducted using pairwise.t.test() in R.

We constructed a tooth morphospace using all four dental topographic metrics analyzed by principal components (PC) analysis. The first and second PC axes were chosen to visualize the 2D morphospace. Additionally, we quantified the degree of morphological disparity and statistical differences in disparity between adjacent time interval data partitions. All PC scores generated from the PCA were included in the disparity analysis. The prcomp() function in R was used for PCA, and the morphol.disparity() function implemented in the Geomorph R package (Adams and Otárola‐Castillo, 2013) was used for morphological disparity significance tests. Input data for the PCA were from the original dataset values, not from the bootstrap samples.

Linear regression analyses between individual dental topographic metrics (DNE, OPCR, Slope, RFI) and dental performance metrics (compressive bite strain energy, shear bite strain energy) were performed to quantify the correlation between dental form and function. Adjusted R^2^ and p-values generated from the lm() function in R were used to evaluate the goodness of fit and statistical significance of the form-function relationships. The distribution of R^2^ values and their corresponding p-values are reported in Figure 3—figure supplement 1.

In addition to pairwise form-function linear regression analyses, we also evaluated the degree of correlation between the DTA and FEA data blocks as a means to measure covariation between dental topography and deformation resistance. We used 2B-PLS analysis (Klingenberg, 2014) coupled with bootstrap resampling to generate distributions of correlation coefficients (*r-pls*) for each of the three time bins, and then tested for significant differences in the magnitude of DTA-FEA correlation between adjacent time bins using Welch’s two-sample t-tests on the correlation coefficient distributions. DTA-FEA correlation differences at p<0.05 are interpreted as a significant shift in the degree of covariation of the two traits from one time bin to the next time bin.

All R scripts used in the analyses are included as supplementary files (Source data 8 and 9).

### Resource availability

- Requests for further information and resources should be directed to and will be fulfilled by the lead contact, Jack Tseng (zjt@berkeley.edu).
- Code associated with analyses is included with this article.
- Raw data are available in the supplementary materials.
- All original fossil specimens are accessioned in the Institute of Vertebrate Paleontology and Paleoanthropology, Chinese Academy of Sciences.

## Data Availability

All data and code are either included as supplemental information or posted at FigShare (https://doi.org/10.6084/m9.figshare.28611854). The following dataset was generated: TsengZJ LiQ TingSY 2026Tooth mesh and finite element models of Paleocene Asian eutherian mammalsfigshare10.6084/m9.figshare.28611854

## Additional files

MDAR checklist

Source data 1. Taxa sampled in the study.

Source data 2. List of specimens used in the study.

Source data 3. Results of finite element simulation convergence tests.

Source data 4. Results of cross-validation between cast and original specimen models used.

Source data 5. Summary of tooth size measurements.

Source data 6. Dental topographic analysis and finite element analysis outputs.

Source data 7. Analysis of variance (ANOVA) results for dental topographic and finite element analyses.

Source data 8. R script for batch processing of dental topography models.

Source data 9. R script for statistical analyses of dental topographic and finite element analyses.

Source data 10. Compilation of Nanxiong Basin paleopalynoflora data.
