# Supplementary material for: Brawn before bite in endemic Asian eutherian mammals after the end-Cretaceous extinction
Source: eLife. 2026 Jun 30;14:RP108917. doi: 10.7554/eLife.108917 (PMC13318306; doi:10.7554/eLife.108917)
Supplement: Peer review history

# Editor report: eLife Assessment

**DOI:** 10.7554/eLife.108917.4.sa0

**Authors:** Sergio Rasmann

**Affiliations:** University of Neuchâtel Switzerland

**Keywords:** Important, Solid

This **important** study fills a major geographic and temporal gap in understanding Paleocene mammal evolution in Asia and proposes an intriguing "brawn before bite" hypothesis grounded in diverse analytical approaches. The work rests on a **solid** methodological base. Some limitations remain, including uncertainty introduced by pooling different tooth positions, limited dietary interpretation, and the predominantly herbivorous taxonomic focus, which narrows the ecological scope of the conclusions. However, the manuscript provides a substantially strengthened and well-supported contribution, while appropriately inviting further work to clarify dietary trends, broader ecological context, and links between dental trait evolution and environmental change.

# Referee report: Reviewer #2 (Public review)

**DOI:** 10.7554/eLife.108917.4.sa1

[Editors' note: this version has been assessed by the Senior Editor without further input from the original reviewers. The authors have addressed the minor comments raised in the previous round of review.]

Summary:

This study uses dental traits of a large sample of Chinese mammals to tract evolutionary patterns through the Paleocene. It presents and argues for a 'brawn before bite' hypothesis -- mammals increased in body size disparity before evolving more specialized or adapted dentitions. The study makes use of an impressive array of analyses, including dental topographic, finite element, and integration analyses, which help to provide a unique insight into mammalian evolutionary patterns.

Strengths:

This paper helps to fill in a major gap in our knowledge of Paleocene mammal patterns in Asia, which is especially important because of the diversification of placentals at that time. The total sample of teeth is impressive and required considerable effort for scanning and analyzing. And there is a wealth of results for DTA, FEA, and integration analyses. Further, some of the results are especially interesting, such as the novel 'brawn before bite' hypothesis and the possible link between shifts in dental traits and arid environments in the Late Paleocene. Overall, I enjoyed reading the paper and I think the results will be of interest to a broad audience.

Weaknesses:

For the original draft of the manuscript, I had four major concerns with the study, especially related to the sampling, diet, and evidence for the 'brawn before bite' hypothesis. I still believe that the original issues that I raised may be weaknesses of the study. For example, there is still limited discussion on diets (even though the dental topographic analyses used in the study are designed for inferring diets). And I find the results a little challenging to interpret because teeth of multiple positions are included in the same samples, which seems problematic. That said, the authors have addressed each of my previous concerns and have made major revisions, including running new analyses, and thus I support the paper.

# Author comment: Author response

**DOI:** 10.7554/eLife.108917.4.sa2

**Authors:** Z Jack Tseng, Qian Li, Suyin Ting

**Affiliations:** University of California, Berkeley Berkeley United States; Institute of Vertebrate Paleontology and Paleoanthropology Beijing China; Natural History Museum of Los Angeles County Los Angeles United States

The following is the authors’ response to the previous reviews

**Public Reviews:**

**Reviewer #2 (Public review):**

Summary:

This study uses dental traits of a large sample of Chinese mammals to tract evolutionary patterns through the Paleocene. It presents and argues for a 'brawn before bite' hypothesis -- mammals increased in body size disparity before evolving more specialized or adapted dentitions. The study makes use of an impressive array of analyses, including dental topographic, finite element, and integration analyses, which help to provide a unique insight into mammalian evolutionary patterns.

Strengths:

This paper helps to fill in a major gap in our knowledge of Paleocene mammal patterns in Asia, which is especially important because of the diversification of placentals at that time. The total sample of teeth is impressive and required considerable effort for scanning and analyzing. And there is a wealth of results for DTA, FEA, and integration analyses. Further, some of the results are especially interesting, such as the novel 'brawn before bite' hypothesis and the possible link between shifts in dental traits and arid environments in the Late Paleocene. Overall, I enjoyed reading the paper and I think the results will be of interest to a broad audience.

Weaknesses:

For the original draft of the manuscript, I had four major concerns with the study, especially related to the sampling, diet, and evidence for the 'brawn before bite' hypothesis. I still believe that the original issues that I raised may be weaknesses of the study. For example, there is still limited discussion on diets (even though the dental topographic analyses used in the study are designed for inferring diets). And I find the results a little challenging to interpret because teeth of multiple positions are included in the same samples, which seems problematic. That said, the authors have addressed each of my previous concerns and have made major revisions, including running new analyses, and thus I support the paper.

This revised submission includes only minor changes aimed at clarifying the main text.

**Reviewer #2 (Recommendations for the authors):**

I appreciate that the authors made many improvements to their study based on reviewers' comments. I don't have any remaining major issues with the paper, but I do have several minor comments.

Thank you for taking the time to provide additional helpful feedback on our study. We have made minor revisions to the manuscript based on your suggestions. Please see our point-by-point response below.

Lines 48-50. I reiterate my suggestion in my previous review to explicitly state which clade is being discussed, which is important because several major mammal groups beyond placentals (metatherians, multituberculates, dryolestoids, gondwanatherians) survived the K-Pg and had very different diversification patterns. You mention "mammal taxonomic diversity" but in the next sentence say "This initial placental mammals diversification ..." and later mention "stem placental/eutherian lineages." To stay consistent, you might replace "mammal" (L48) and "placental mammals" (L50) with "eutherian(s)" (usually defined as stem + crown placentals). If you follow this suggestion, then elsewhere in the paper I recommend replacing "mammals" with "eutherians" for consistency.

Thank you for this suggestion. We modified the use of “mammals” throughout the text to general reference to the group only; specific mentions of the dataset analyzed are revised to “eutherians.”

Lines 75-83. I respect the authors' hesitancy to reconstruct specific diets for the fossil taxa (L75-83), especially considering that dental topographic analyses (DTAs) often struggle to differentiate diets in extant taxa (e.g., Pineda-Munoz et al. 2016 Methods Ecol Evol). I still think that the authors might be able to interpret dietary trends from their results (e.g., an increase in average OPCR values indicating a shift toward more herbivorous diets) - I think discussing dietary trends would be an interesting discussion topic later in the paper. That said, I also recognize that different DTA results seem to show conflicting dietary trends (based on my limited knowledge of those metrics) so maybe that complicates things too much.

We concur with Reviewer 2 that dietary inferences of DTA data are premature, especially given the ongoing controversies of its use in studies of extant mammal teeth. We kept our current scope of discussion unchanged.

Lines 75-77. "early mammals ... are beyond the reach of conventional phylogenetic bracketing approaches to dietary reconstruction." But your fossils (eutherians) are certainly within 'phylogenetic brackets' of modern clades (therians, i.e. Eutheria + Metatheria). Maybe you're alluding to the fossils being stem lineages of extant subgroups like Ungulata, which means we can't bracket them specifically within those eutherian subgroups? So, I recommend revising or expanding your statement for clarity. Also, the considerable phylogenetic uncertainty for Paleocene groups (e.g., Halliday et al. 2015) complicates this issue, which you could mention.

We modified the sentence to now say “Additional complications with ecomorphological analysis of these stem eutherians include the uncertainty in their dietary ecology, having diverged prior to the crown radiation, and uncertainty in phylogenetic positions of Paleocene taxa [7]; thus, they are beyond the reach of conventional phylogenetic bracketing approaches to dietary reconstruction.”

Line 84. "We investigated dental topography-performance shifts ...". You haven't introduced dental topography or even mentioned teeth yet, and "performance shifts" is vague. So, this phrase might confuse readers. Maybe you can just erase it and start the sentence with "We investigated the timing of ecomorphological ..."?

We made the recommended revision.

Lines 104-105 (and elsewhere). "Dental traits paralleled Paleocene global and regional environmental conditions" and "We found that dental topographic trait variability in Paleocene mammals in south China tracked global and regional climatic changes". These conclusions seem a little too assertive to me. Your sample is grouped into 3 rough time bins (of somewhat uncertain ages) and is from a relatively small geographic range - that seems like very limited information for inferring links between dental patterns and climatic changes, especially global patterns. I think it's worth HYPOTHESIZING that dental traits are linked to environmental/climatic changes (with results like those in Figure 2A & B as evidence to support that hypothesis), but I wouldn't make that claim with any confidence. So, I recommend that you temper your relevant conclusion statements. For example, for Line 105, you could replace "We found ..." with "We posit ..." (L105). I would make similar changes to similar statements throughout the paper (e.g., L243).

Thank you for this suggestion to temper our phrasing. We edited throughout the text to make our interpretations less assertive.

Figure 1 (and your response to reviewers). Why was the timescale changed to 65.5 Ma for the K-Pg boundary? The K-Pg is 66 Ma (not 65.5), which is the age you mention in the text (e.g. Pg 3 L39) and is well established in the literature - see recent papers from the Paul Renne lab for a more exact age.

We revised the figure to have the K-Pg at 66 Ma.
